# NFEmbed: modeling nitrogenase activity via classification and regression with pretrained protein embeddings

**DOI:** 10.1093/bioadv/vbaf204

**Published:** 2025-08-23

**Authors:** Md Muhaiminul Islam Nafi, Abdullah Al Mohaimin

**Affiliations:** Department of CSE, BUET, Dhaka 1000, Bangladesh; Department of CSE, United International University (UIU), Dhaka 1212, Bangladesh; Department of CSE, BUET, Dhaka 1000, Bangladesh

## Abstract

**Motivation:**

Heavy usage of synthetic nitrogen fertilizers to satisfy the increasing demands for food has led to severe environmental impacts like decreasing crop yields and eutrophication. One promising alternative is using nitrogen-fixing microorganisms as biofertilizers, which use the nitrogenase enzyme. This could also be achieved by expressing a functional nitrogenase enzyme in the cells of the cereal crops.

**Results:**

In this study, we predicted microbial strains with a high potential for nitrogenase activity using machine learning techniques. Its objective was to enable the screening and ranking of potential strains based on genomic information. We explored several protein language model embeddings for this prediction task and built two stacking ensemble models. One of them, NFEmbed-C, used k-Nearest Neighbors and Random Forest as base and meta learners, respectively. The other one, NFEmbed-R, combined Decision Tree Regressor and eXtreme Gradient Boosting Regressor as base learners, with Support Vector Regressor as the meta learner. On the Test set, both NFEmbed-C and NFEmbed-R performed better than the state-of-the-art methods with improvements ranging from 0% to 11.2% and from 30% to 51%, respectively. While NFEmbed-R got a 0.783 *R*^2^ score, 0.158 MSE, and 0.398 RMSE, NFEmbed-C acquired 0.949 sensitivity, 0.892 F1 score, and 0.784 Matthews Correlation Coefficient on the test set.

**Availability and implementation:**

We performed our analysis in Python; code is available at https://github.com/nafcoder/NFEmbed

## 1 Introduction

As highlighted by the 2023 State of Food Security and Nutrition in the World report, which was jointly published by five United Nations agencies, global food security remains a pressing concern. The report makes an estimation that about 733 million people faced hunger in 2023. That represents 1 in 11 individuals worldwide, and worryingly, 1 in 5 individuals in Africa (UNICEF 2024). Climate change, which is continuously disrupting agricultural productivity through unpredictable weather patterns, soil degradation, and increased pest prevalence, is exacerbating the growing food crisis. To alleviate the rapidly increasing demands of food for a growing global population, synthetic nitrogen fertilizers have been heavily used in modern agriculture. Not only did these fertilizers decrease the crop yields in the long run, but their overuse and inefficiency have also led to severe environmental impacts like global warming and eutrophication. So, an alternate approach is needed that will help in increasing food production and also not affect the climate in a harmful way. One promising option is using nitrogen-fixing microorganisms as biofertilizers. Certain bacteria and Archaea, commonly known as the diazotrophs, have the enzyme nitrogenase, which allows them to fix nitrogen directly from the atmosphere. This organic process provides a biological pathway to augment or even replace synthetic fertilizers and is a fundamental component of the global nitrogen cycle. By either transferring the ability to establish a symbiotic relationship with nitrogen-fixing bacteria or by expressing a functional nitrogenase enzyme in the cells of the cereal crop (Rogers and Oldroyd 2014), we can engineer cereal crops with the ability to fix nitrogen on their own.

A metal-rich complex called nitrogenase is the main enzyme in charge of biological nitrogen fixation. There are currently three identified homologous nitrogenase isoforms. All known diazotrophs contain molybdenum (Mo) nitrogenase (encoded by nifHDK), the most common and well-studied nitrogenase. When Mo is depleted, some diazotrophs express “back-up” or alternative nitrogenase genes, such as vnfHDGK for vanadium (V) nitrogenase or anfHDGK for iron (Fe) nitrogenase (Schmidt *et al.* 2024). The overall nitrogen fixation reaction catalyzed by molybdenum nitrogenase can be summarized as follows:

(1) $$N_{2}{+8H}^{+}{+8e}^{-}+16\text{ATP}\to {2\text{NH}}_{3}{+H}_{2}{+16\text{ADP}+16P}_{i}$$

The mechanism of nitrogenase is complicated and still not fully clear even after decades of research. The enzyme catalyzes a multielectron reduction of nitrogen gas to ammonia. This reaction is tightly regulated and energetically expensive. The activity of nitrogenase is most commonly assayed using the acetylene reduction assay (ARA) (Dilworth 1966). One of the major challenges in using nitrogenase is its extreme sensitivity to oxygen. Oxygen inactivates the enzyme and necessitates protective adaptations within the host organism (Alleman and Peters 2023).

Recent studies (Zhang *et al.* 2023, Peng *et al.* 2024, Ye *et al.* 2024) have been focusing on systems biology and computational models to understand the regulatory and metabolic networks governing nitrogenase expression and activity. These studies mainly aim to reveal the minimal functional gene sets and critical biochemical pathways required for efficient nitrogen fixation. Recently, Ye *et al.* (2025) built a machine learning (ML) model named Carmna to predict the nitrogenase activity values from a given species strain. The classification version of Carmna predicted whether the given strain would exhibit nitrogenase activity more than 50 nmol C_2_H_4_/mg protein/hour or not. And, the regression version of Carmna predicted the nitrogenase activity scores. Carmna (classification) was an eXtreme Gradient Boosting (XGB) (Chen and Guestrin 2016) model, and Carmna (regression) was a stacking ensemble model where the base and meta learners were Support Vector Regressor (SVR) (Drucker *et al.* 1997) models. As features for the ML model, Carmna used ProtT5-XL-U50 (Elnaggar *et al.* 2022) protein language model (PLM) embeddings, protein sequence-based features, gene expression, gene distance, and codon preference, etc.

But some limitations of Carmna exist and are yet to be addressed. Ye *et al.* did not utilize different PLM embeddings in their study. PLM embeddings have emerged to be an effective feature in many different prediction tasks (Kim and Kwon 2023, Akbar *et al.* 2024, Dang and Vu 2024, Mishra *et al.* 2024, Simon and Bankapur 2024, Sun *et al.* 2024, Ullah *et al.* 2024, Akbar *et al.* 2025, Shahid et al. 2025) in recent years. In our study, we explored different PLM embeddings like ProtT5-XL-U50 (Elnaggar *et al.* 2022) embeddings, ProteinBERT (Brandes *et al.* 2022) embeddings, and ESM C (EvolutionaryScale Team 2024) embeddings, etc. to predict nitrogenase activity. Also, Carmna did not use feature selection. We utilized Incremental Feature Selection (IFS) in our study to properly choose the optimal feature set for both classification and regression tasks. Additionally, Carmna did not explore the stacking ensemble approach for the classification task and did not fully utilize the stacking ensemble approach for the regression task. But we thoroughly developed the stacking ensemble model for both tasks.

In this article, we used ML techniques to identify microbial strains and species that have a high potential for nitrogenase activity. Our goal was to aid in the development of biofertilizers using the nitrogen-fixing ability of the diazotrophs. We proposed two stacking ensemble models in this study. NFEmbed-C is used for classification and NFEmbed-R is used for regression. First, we experimented with various PLM embeddings and determined that the ESMC_600M PLM embeddings were the best fit for the prediction tasks. To choose the best feature group combination for the two proposed models, we then applied IFS to the feature groups. Several regression and classification models were examined for the model selection process, and the best base and meta learners were selected. We chose the base learners based on the Incremental Mutual Information (IMI) approach’s Mutual Information (MI) scores. The five-fold cross-validation (CV) performance results were then used to select the meta learners. NFEmbed-C used k-Nearest Neighbors (KNN) (Peterson 2009) as the base learner and Random Forest (RF) (Breiman 2001) as the meta learner. In contrast, NFEmbed-R employed Decision Tree Regressor (DTR) (Breiman *et al.* 2017) and eXtreme Gradient Boosting Regressor (XGBR) (Chen and Guestrin 2016) as base learners, with SVR (Drucker *et al.* 1997) serving as the meta learner. To illustrate the efficacy of the proposed models, we included regression plots, t-distributed Stochastic Neighbor Embedding (t-SNE) (Van der Maaten and Hinton 2008), and Uniform Manifold Approximation and Projection (UMAP) (McInnes *et al.* 2018) visualizations on the feature space. Additionally, a Shapley Additive Explanations (SHAP) (Scott *et al.* 2017) interoperability analysis was conducted to offer some biological contexts for our proposed models’ predictions. The relative significance of distinct genes and amino acid (AA) residues in promoting enzymatic activity was also evaluated using SHAP. Our method improves our comprehension of the molecular elements influencing nitrogenase efficiency, in addition to making it possible to screen and rank candidate strains from genomic data.

The key contributions of this study are given below.

- NFEmbed-C and NFEmbed-R both outperformed state-of-the-art methods on the Test set. NFEmbed-C got a sensitivity of 0.949, balanced accuracy of 0.891, F1 score of 0.892, and Matthews Correlation Coefficient (MCC) of 0.784 on the test set, while NFEmbed-R got a 0.783 *R*^2^ score (coefficient of determination), 0.158 mean squared error (MSE), 0.398 root mean squared error (RMSE), and 0.234 mean absolute error (MAE).
- We used five-fold CV and the IFS approach for feature selection.
- We employed five-fold CV and the IMI approach for model selection.
- SHAP analysis was employed to evaluate the selected feature groups and demonstrate the effectiveness of the stacking ensemble.
- SHAP analysis was also used to assess the relative importance of various genes and AA residues in fostering enzymatic activity.

The remaining sections are arranged as follows. The materials and techniques used in this article are described in Section 2. The results of several experiments, a performance assessment, and a comparison of our methodology are shown in Section 3. The implications of our findings in relation to nitrogenase activity prediction are examined in Section 4, which also concludes the article with possible future directions.

## 2 Methods

In this section, descriptions about the dataset, feature groups, feature selection, model selection, and configurations of the final model are provided.

### 2.1 Dataset

We used the same dataset used to develop Carmna, which was collected by Ye *et al.* (2025). They compiled nitrogenase activity values from the literature that were primarily measured using the ARA (He *et al.* 2024). For this, they conducted an extensive search via Google Scholar. All nitrogenase activity values were standardized to units of nmol C_2_H_4_/mg protein/hour to maintain consistency. For multiple activity values for a particular sample, they took the median value. They also retrieved Genomic data for nitrogen-fixing bacteria and their corresponding nitrogenase protein sequences from the NCBI (Schoch *et al.* 2020) database. From this process, they collected a final dataset containing 402 samples from various nitrogenase-producing strains. To classify enzyme activity, they applied a threshold of 50 nmol C_2_H_4_/mg protein/hour. The activity values higher than this threshold were considered as positive (high activity) samples, and those lower than this threshold were considered as negative (low activity) samples. Thus, the dataset included 209 negative and 193 positive samples. With stratified sampling, they allocated 80% of the data to the Training set (167 negative and 154 positive samples) and the remaining 20% to the Test set (42 negative and 39 positive samples). We further divided the training set into two parts: 60% as the Training-60 set and the remaining 40% as the Training-40 set for building the stacking ensemble model.

For the regression task of predicting the nitrogenase activity values, they applied a logarithmic transformation (given below) to the activity values to reduce the impact of extreme labeling gaps.

(2) $$y={\text{log}}_{10}(x+2)$$

In the above equation, *x* is the actual activity value, and *y* is the logarithmically transformed regression label.

### 2.2 Feature extraction

In this subsection, different feature groups used in this study are described.

#### 2.2.1 Composition of triplets

Twenty AAs were clustered into seven classes based on their side-chain dipole moments and volumes (Shen *et al.* 2007, Sun *et al.* 2017), capturing local sequence patterns based on chemical properties. Then, the frequencies of all possible triplets of these classes within each protein sequence encoded by the nifH, nifD, and nifK genes belonging to a particular sample were calculated, resulting in a 343-dimensional (7 × 7 × 7) feature vector (Sun *et al.* 2017) for that sample. The triplet (CT) frequencies were then averaged across the three protein sequences for each sample. This feature group encodes the compositional distribution of chemical environments in the proteins. The feature dimension is 343.

#### 2.2.2 Dipeptide composition

The interactions between AA residues at various positions are captured by dipeptide composition (DPC) (Bhasin and Raghava 2004, Liu *et al.* 2010, Du *et al.* 2017). Initially, the residue of each protein sequence encoded by the nifH, nifD, and nifK genes was given a 20×20 matrix, which was then flattened to obtain a feature group size of 400. The values were averaged across the three protein sequences for each sample. The number of dimensions in this feature group is 400.

#### 2.2.3 Pseudo-AA composition

Instead of capturing the frequency of each AA (20 dimensions), pseudo-AA composition (PAAC) incorporates additional information about the sequence order and physicochemical properties (e.g. hydrophobicity, polarity, and side-chain mass). This reflects both composition and local sequence patterns (Chou 2005, Zhang *et al.* 2021). Here, 50-dimensional PAACs were generated for each protein sequence encoded by the nifH, nifD, and nifK genes belonging to a particular sample. Among the 50 features, 20 corresponded to the standard AA composition, and 30 captured pseudo components that reflect the influence of physicochemical correlations between AAs at different positions. The 20 AAs were the following: Alanine (Ala), Cysteine (Cys), Aspartic Acid (Asp), Glutamic Acid (Glu), Phenylalanine (Phe), Glycine (Gly), Histidine (His), Isoleucine (Ile), Lysine (Lys), Leucine (Leu), Methionine (Met), Asparagine (Asn), Proline (Pro), Glutamine (Gln), Arginine (Arg), Serine (Ser), Threonine (Thr), Valine (Val), Tryptophan (Trp), and Tyrosine (Tyr). The values were averaged across the three protein sequences for each sample. This is a 50-dimensional feature group.

#### 2.2.4 Relative synonymous codon usage

Relative synonymous codon usage (RSCU) (Orešič and Shalloway 1998) measures the relative usage frequency of synonymous codons, capturing codon preference patterns within a species. RSCU was calculated for 61 sense codons (excluding the three stop codons) for each protein sequence encoded by the nifH, nifD, and nifK genes. The values were averaged across the three protein sequences for each sample. The feature dimension is 61.

#### 2.2.5 Euclidean distance

Euclidean distance (euclidean_distance) (Wang *et al.* 2024) usually quantifies the straight-line distance between two vectors in a multidimensional space. The RSCU values were used for calculating the Euclidean distance. To measure the deviation from a neutral codon usage, Euclidean distance was computed between the target species and a reference species with no codon bias (where all codons have an RSCU value of 1). The equation to calculate it is given below. This is a one-dimensional feature.

(3) $$\text{ECD}=\sqrt{\sum_{i=1}^{n}{\left(x_{i}-1\right)}^{2}}$$

Here, *n* is the total number of codons, and $x_{i}$ is the RSCU value of the *i*th codon in the target species.

#### 2.2.6 Expression

The Codon Adaptation Index (CAI) (Sharp and Li 1987) was computed using the CAI Python package (v1.0.3) (Lee 2018). It indicates how closely a gene’s codon usage aligns with that of highly expressed genes. It was computed as the geometric mean of the relative synonymous codon usage (RSCU) values of the gene’s codons, normalized by the preferred codons in the reference set. E-values and Fop-values (frequency of optimal codons) (Ikemura 1981, Karlin and Mrázek 2000) were calculated using the coRdon R package (v1.22.0) (Elek *et al.* 2018). E-values reflect deviations from uniform codon usage within synonymous families, while Fop-values represent the proportion of optimal codons used. In total, CAI, E-, and Fop-values were calculated for nifA, nifB, nifD, nifE, nifH, nifK, nifN, and nifX genes. So, the feature dimension is 24 (8 × 3).

#### 2.2.7 Gene distance

We used the intragenomic distance (gene_distance) (how far apart these three genes are located from one another) between the nifH, nifD, and nifK genes that capture potential regulatory implications of their genomic organization. This is another one-dimensional feature.

#### 2.2.8 Copy number

We used the gene counts of 34 different genes (copy_number) (nifD, nifH, nifK, amtB, fixA, fixB, fixC, fixX, glnK, glnA, nifA, nifB, nifE, nifF, nifJ, nifL, nifM, nifN, nifP, nifQ, nifS, nifT, nifU, nifV, nifW, nifX, nifY, nifZ, rnfA, rnfB, rnfC, rnfD, rnfE, and rnfG) within the genome. So, the feature dimension is 34.

#### 2.2.9 ProtT5-XL-U50 embeddings

Using 393 billion AAs of UniRef and BFD data, the authors of ProtTrans (Elnaggar *et al.* 2022) examined two autoregressive models (Transformer-XL, XLNet) and four auto-encoder models (BERT, Albert, Electra, T5) (ProtT5-XL-U50). From their work, ProtT5-XL-BFD, ProtT5-XL-UniRef50 (or ProtT5-XL-U50), ProtT5-XXL-BFD, ProtLNet, ProtAlbert, ProtBert, ProtBert-BFD, ProtElectra-Discriminator-BFD, and ProtElectra-Generator-BFD are the 10 models that are available. The most effective of them in the TS115, CASP12, DeepLoc, and CB513 benchmarks was ProtT5-XL-U50. So, we used ProtT5-XL-U50 embeddings in our study. The size of this embedding is 1024. To extract the features, we used the default parameter settings provided in the official ProtTrans GitHub repository. We took the per-residue embeddings from ProtT5-XL-U50 and averaged along the sequence length axis to obtain global embeddings for each protein encoded by the nifH, nifD, and nifK genes. These global embeddings were then averaged across the three protein sequences for each sample. So, the final feature dimension is 1024.

#### 2.2.10 ProteinBERT embeddings

ProteinBERT (ProteinBERT) (Brandes *et al.* 2022) was developed to naturally capture local and global representations of proteins. Approximately 106 million proteins from the UniProtKB/UniRef90 dataset were used to train this model. The global embedding size is 512. To extract the features, we used the default parameter settings provided in the official ProteinBERT GitHub repository. We took the global embeddings from ProteinBERT for each protein encoded by the nifH, nifD, and nifK genes. These global embeddings were then averaged across the three protein sequences for each sample. So, the number of features in this group is 512.

#### 2.2.11 Ankh embeddings

Elnaggar *et al.* (2023) developed a protein-specific optimized language model by systematically evaluating over 20 architectural and training configurations. They used UniRef50 as the pretraining dataset. Two models (Ankh and Ankh base) were released by them. Ankh achieved state-of-the-art results across a range of structure and function prediction benchmarks (e.g. CASP12, CASP14, ProteinNet, DeepLoc, and GB1). We used the Ankh model in our study, which has an embedding size of 1536. To extract the features, we used the default parameter settings provided in the official Ankh GitHub repository. To get global embeddings for every protein encoded by the nifH, nifD, and nifK genes, we averaged the per-residue embeddings from Ankh along the sequence length axis. For every sample, these global embeddings were then averaged over the three protein sequences. Thus, 1536 is the final feature dimension.

#### 2.2.12 ESM C model embeddings

ESM Cambrian (ESM C) (ESMC_300M, ESMC_600M and ESMC_6B) (EvolutionaryScale Team 2024) is a next-generation PLM developed by the ESM team. It was designed as a parallel model family to their flagship generative models (ESM-3; Hayes *et al.* 2025). While the ESM-3 model focuses on controllable protein sequence generation, ESM C was optimized for learning biologically meaningful representations through unsupervised learning. It was trained on large-scale protein sequence datasets clustered from UniRef, MGnify, and the Joint Genome Institute (JGI). It totaled billions of clusters at 70% sequence identity. Three different ESM C models were released, having 300M, 600M, and 6B parameters. We named them ESMC_300M, ESMC_600M, and ESMC_6B model, respectively. Their embedding sizes were 960, 1152, and 2560, respectively. Along the sequence length axis, we averaged the per-residue embeddings from the ESMC_300M, ESMC_600M, and ESMC_6B models to obtain global embeddings for each protein encoded by the nifH, nifD, and nifK genes. The three protein sequences were then averaged across these global embeddings for each sample. Consequently, the final feature dimensions of the ESMC_300M, ESMC_600M, and ESMC_6B models were 960, 1152, and 2560, respectively. To extract the features from ESMC_300M and ESMC_600M, we used the default parameter settings provided in the official ESM GitHub repository. For extracting ESMC_6B embeddings, we used Forge API.

### 2.3 Feature selection

We used IFS to select features for both classification and regression tasks. We ran IFS on the feature groups (described in Section 2.2) as follows.

The first step is to select the feature group with the highest five-fold CV F1-score using an evaluation model. We then attempt to measure the prediction performance by adding another feature group to it. Out of all feature group pairs that are thus produced, the pair with the best five-fold CV F1-score is retained. Similarly, we now attempt to add another feature group to this pair. In this manner, we greedily explored a subset of potential feature group combinations to find the best one rather than thoroughly examining every one of them. Because XGB (Chen and Guestrin 2016) has consistently shown superior predictive capabilities across a variety of research fields (Grinsztajn *et al.* 2022, Bohacek and Bravansky 2024, Uddin *et al.* 2024), it was chosen as the evaluation model to perform IFS for the classification task. The optimal feature group combination for the classification task turned out to be “ESMC_600M, CT, RSCU, Copy_number, Gene_distance” (see Results).

**Figure 1.**
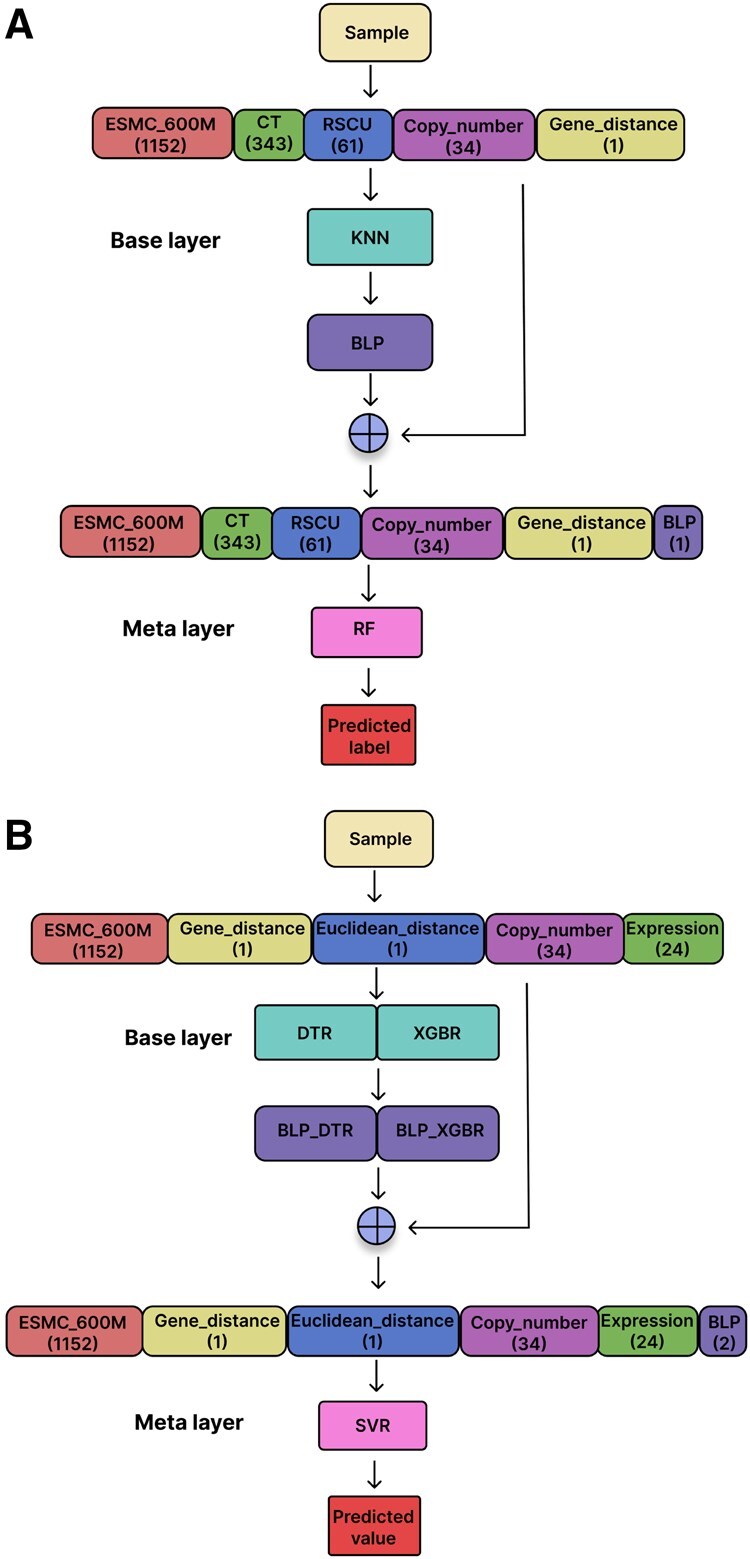
The prediction framework for our proposed models. A) Prediction framework for the NFEmbed-C model (classification). B) Prediction framework for the NFEmbed-R model (regression).

The same IFS algorithm is applied to the regression task. The sole modification is that it now uses *R*^2^ scores rather than F1-scores. Since XGBR (Chen and Guestrin 2016) has also been used as a highly capable regression model in many research fields (Shahani *et al.* 2021, Kıyak *et al.* 2024), it is used as the evaluation model. “ESMC_600M, Gene_distance, Euclidean_distance, Copy_number, Expression” was the best feature group combination for the classification task (see Results). The optimal feature group combinations for our proposed classification and regression models can be seen in Fig. 1A and B, respectively.

### 2.4 Performance evaluation

A well-established set of classification performance metrics (Altman and Bland 1994, Fawcett 2006, Powers 2011) was used to assess the model’s ability to predict the binary labels: F1 score (F1), area under the curve (AUC), sensitivity (SN), specificity (SP), accuracy (ACC), balanced accuracy (BACC), precision (PREC), and MCC. Below are the equations used to determine these scores. True positives, false positives, true negatives, and false negatives are denoted by TP, FP, TN, and FN, respectively, in this context.

(4) $$F1=\frac{2\text{TP}}{2\text{TP}+\text{FP}+\text{FN}}$$

 

(5) $$\text{SN}=\frac{\text{TP}}{\text{TP}+\text{FN}}$$

 

(6) $$\text{SP}=\frac{\text{TN}}{\text{TN}+\text{FP}}$$

 

(7) $$\text{ACC}=\frac{\text{TP}+\text{TN}}{\text{FP}+\text{TP}+\text{TN}+\text{FN}}$$

 

(8) $$\text{BACC}=\frac{\text{SN}+\text{SP}}{2}$$

 

(9) $$\text{PREC}=\frac{\text{TP}}{\text{FP}+\text{TP}}$$

 

(10) $$\text{MCC}=\frac{\left(\text{TP}\times \text{TN})-(\text{FP}\times \text{FN}\right)}{\sqrt{\left(\text{TP}+\text{FN})\times (\text{TP}+\text{FP})\times (\text{TN}+\text{FP})\times (\text{TN}+\text{FN}\right)}}$$

To assess the prediction ability of the model, a similar set of well-established regression performance metrics (Willmott and Matsuura 2005, Pedregosa *et al.* 2011, Chicco *et al.* 2021) was utilized: coefficient of determination (*R*^2^), MSE, RMSE, and MAE. Below are the equations used to determine these scores. The true value, predicted value, and mean of the true values across *n* samples are denoted by $y_{i}$, ${\hat{y}}_{i}$, and $\bar{y}$, respectively, in this context.

(11) $$R^{2}=1-\frac{\sum_{i=1}^{n}{\left(y_{i}-{\hat{y}}_{i}\right)}^{2}}{\sum_{i=1}^{n}{\left(y_{i}-\bar{y}\right)}^{2}}$$

 

(12) $$\text{MSE}=\frac{1}{n}\sum_{i=1}^{n}{\left(y_{i}-{\hat{y}}_{i}\right)}^{2}$$

 

(13) $$\text{RMSE}=\sqrt{\frac{1}{n}\sum_{i=1}^{n}{\left(y_{i}-{\hat{y}}_{i}\right)}^{2}}$$

 

(14) $$\text{MAE}=\frac{1}{n}\sum_{i=1}^{n}|y_{i}-{\hat{y}}_{i}|$$

### 2.5 Model description

We built a stacking ensemble with two learner phases—referred to as *base learners* in the base layer and a single *meta learner* in the meta layer. Each learner captures distinct patterns and relationships, and base learners are separate ML models that are trained independently on the dataset. The model known as the meta learner uses the initial input features as well as the predictions made by the base learners as inputs. The final prediction is improved by the meta learner by incorporating knowledge from both sources. The Training set was used to train two distinct ensembles for classification and regression tasks using the same methodology. The base learners were trained using the Training-60 set, and the meta learner was trained using the Training-40 set. The best feature group combination identified by the IFS method (described in Section 2.3) was used as features for the data. The probabilities derived from the base layer, which we referred to as the Base Learner Probabilities (BLP) vector, were used to augment the feature vectors for Training-40 during the meta layer training process.

### 2.6 Model selection

Ten traditional ML models were explored in this study for classification purposes. They were the following: RF (Breiman 2001), XGB (Chen and Guestrin 2016), KNN (Peterson 2009), Gaussian Process Classifier (GPC) (Williams and Rasmussen 2006), Light Gradient Boosting Machine (LBM or LightGBM) (Ke *et al.* 2017), SVM (Cortes 1995), Adaptive Boosting (ADA or AdaBoost) (Freund and Schapire 1997), Gradient Boosting Classifier (GBC) (Friedman 2001), Multilayer Perceptron (MLP) (Rumelhart *et al.* 1985), Quadratic Discriminant Analysis (QDA) (Fisher 1936). For the regression task, we explored nine different ML models, which were the following: XGBR (Chen and Guestrin 2016), k-Nearest Neighbors Regressor (KNNR) (Altman 1992), Gradient Boosting Regressor (GBR) (Friedman 2001), Random Forest Regressor (RFR) (Breiman 2001), SVR (Drucker *et al.* 1997), Least Absolute Shrinkage and Selection Operator (Lasso) (Tibshirani 1996), Ridge Regression (Ridge) (Hoerl and Kennard 1970), Bayesian Ridge Regression (BR) (MacKay 1992), DTR (Breiman *et al.* 2017). Using MI (Vergara and Estévez 2014) between learner outputs and the actual labels (for classification task) or activity values (for regression task), the IMI approach was used to select base learners. IFS and IMI operate similarly. The sole distinction is that IMI looks for the best group of learners rather than the best feature group combination, and the objective function is MI rather than F1-score or *R*^2^ score. KNN was chosen as the base learner for the classification task, and DTR and XGBR for the regression task as a result of IMI. On the other hand, RF was chosen as the meta learner for the classification task and SVR for the regression task since they were the best individual models in terms of five-fold CV predictive performance (see Section 3). The prediction framework for our proposed classification and regression models are illustrated in Fig. 1.

### 2.7 Hyperparameter tuning

To tune the hyperparameters of the chosen base and meta learners for both classification and regression, we employed Grid Search. Table 1 reports the grids that were searched and the values that were ultimately chosen for each learner. The “Tuned value” column presents the specific values selected during hyperparameter optimization for each model. Parameters not explicitly listed were used with their default settings as defined in the Scikit-learn (sklearn) Python package, version 1.2.2.

**Table 1.** Hyperparameters and final selected (tuned) values for each learning model.

Task	Layers	Learners	Hyperparameters	Searched values	Tuned value
Classification	Base layer	KNN	n_neighbors	3, 5, 7	5
			weights	Uniform, distance	Uniform
			algorithm	Auto, ball_tree, kd_tree, brute	Auto
			leaf_size	30, 50, 100	30
	Meta layer	RF	n_estimators	100, 200	200
			max_depth	None, 10	None
			min_samples_split	2, 5	5
			min_samples_leaf	1, 2	1
			max_features	sqrt, log2, None	log2
			bootstrap	True, False	True
			threshold	0.4, 0.45, 0.5, 0.55, 0.6, 0.65, 0.7	0.55
Regression	Base layer	DTR	max_depth	None, 5, 10, 20	5
			splitter	Best, random	Random
			min_samples_split	2, 5, 10	10
			min_samples_leaf	1, 2, 4	1
			max_features	sqrt, log2, None	sqrt
		XGBR	max_depth	3, 5, 10	5
			learning_rate	0.01, 0.1, 0.2	0.01
			n_estimators	100, 200	200
			gamma	0, 1, 5	0
	Meta layer	SVR	kernel	rbf, linear	5
			C	0.1, 1, 10	1
			epsilon	0.01, 0.1, 1	0.01
			gamma	Scale, auto	Scale

## 3 Results

In this section, the results of the experiments conducted in this study are discussed.

### 3.1 Feature selection results

**Figure 2.**
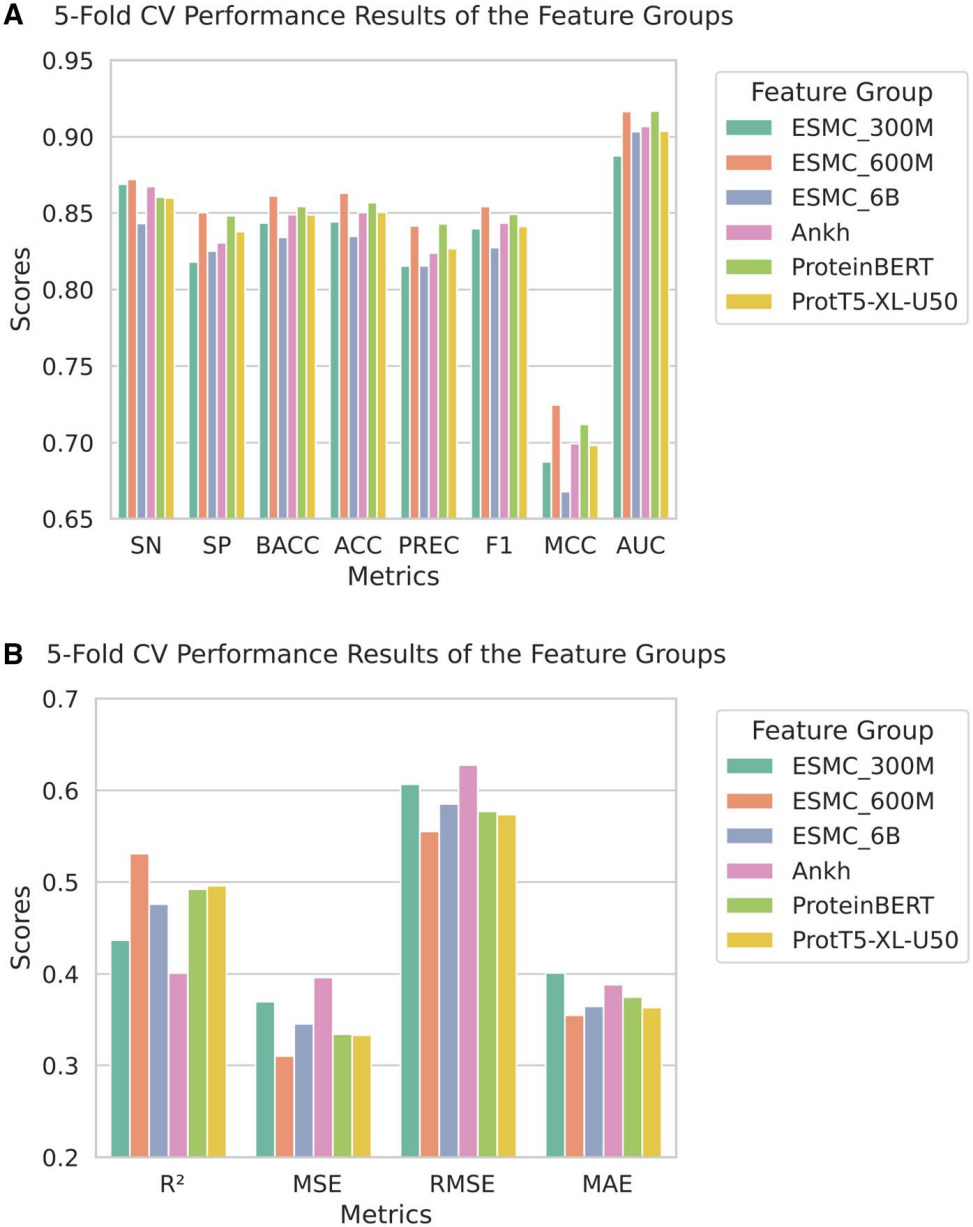
First step of feature selection for classification and regression tasks. A) Five-fold CV performance of different XGB models using six distinct protein language model embeddings. All models were trained on the Training set (classification). B) Five-fold CV performance of different XGBR models using six distinct protein language model embeddings. All models were trained on the Training set (regression).

Initially, we trained separate XGB and XGBR models using the PLM embeddings described in Section 2.2 for the classification and regression tasks, respectively. XGB and XGBR models were used as they have continuously shown superior predictive capabilities across a variety of research fields (Shahani *et al.* 2021, Grinsztajn *et al.* 2022, Bohacek and Bravansky 2024, Kıyak *et al.* 2024, Uddin *et al.* 2024). We then compared their performance in Fig. 2. In Fig. 2A, the five-fold CV performance results of different XGB models trained with various PLM embeddings are presented. It shows that the ESMC_600M embeddings outperformed others across most performance metrics. ESMC_600M embeddings showed better SN, SP, BACC, ACC, F1, and MCC scores than other PLM embeddings. The reported values represent the average performance over all folds. Similarly, Fig. 2B compares the five-fold CV performance of different XGBR models trained using different PLM embeddings, where ESMC_600M embeddings consistently performed best across all metrics (*R*^2^, MSE, RMSE, and MAE). Therefore, for both classification and regression tasks, ESMC_600M embeddings were selected as the final PLM embeddings for our proposed model. To ensure more reliable and robust predictive outcomes, we repeated the five-fold CV procedure 30× using different random seeds and averaged the results for both tasks. The outcomes, presented in Supplementary Fig. 1A and B, available as supplementary data at *Bioinformatics Advances* online, remained similar to the previous single five-fold CV results, confirming the generalizability of our findings.

As described in Section 2.3, we did IFS for both classification and regression tasks, and their results are presented in Tables 2 and 3, respectively. In Table 2 (for classification), XGB models were trained on different feature combinations, and their five-fold CV performance results are presented. The reported values represent the average performance over all folds. Among them, the XGB model trained using the feature combination “ESMC_600M, CT, RSCU, Copy_number, Gene_distance” had the highest F1 score (0.875). Thus, for our classification model, this feature combination was finalized. Similarly, in Table 3 (for regression), five-fold CV performance results of XGBR models trained using different feature combinations are presented. The XGBR model trained using the “ESMC_600M, Gene_distance, Euclidean_distance, Copy_number, Expression” had the highest *R*^2^ score (0.531). Therefore, this feature combination was finalized for our regression model. We averaged the results for both tasks after repeating the five-fold CV process 30× with various random seeds to guarantee more dependable and robust predictive results. Our findings were confirmed to be generalizable as the results, which are presented in Supplementary Tables 1 and 2, available as supplementary data at *Bioinformatics Advances* online, stayed consistent with the single five-fold CV results.

**Table 2.** Classification: five-fold CV performance of XGB model, trained on the Training set, using IFS with different feature combinations.

Feature set	SN	SP	BACC	ACC	PREC	F1^a^	MCC	AUC
Copy_number	0.787	0.719	0.753	0.757	0.713	0.747	0.51	0.831
CT	0.833	0.842	0.837	0.838	0.821	0.825	0.674	0.902
DPC	0.832	0.852	0.842	0.847	0.846	0.834	0.693	0.91
Euclidean_distance	0.696	0.706	0.701	0.698	0.696	0.684	0.407	0.776
Expression	0.801	0.831	0.816	0.819	0.812	0.806	0.633	0.881
Gene_distance	0.639	0.627	0.633	0.629	0.614	0.621	0.265	0.739
PAAC	0.843	0.857	0.85	0.85	0.841	0.84	0.699	0.903
RSCU	0.797	0.841	0.819	0.819	0.814	0.804	0.635	0.89
ESMC_600M	0.872	0.85	0.861	0.863	0.842	0.854	0.725	0.917
ESMC_600M, Copy_number	0.872	0.859	0.866	0.869	0.853	0.861	0.735	0.918
ESMC_600M, CT	0.885	0.844	0.865	0.869	0.848	0.864	0.736	0.909
ESMC_600M, DPC	0.874	0.817	0.846	0.847	0.817	0.842	0.694	0.91
ESMC_600M, Euclidean_distance	0.866	0.872	0.869	0.872	0.863	0.862	0.741	0.916
ESMC_600M, Expression	0.86	0.824	0.842	0.844	0.819	0.837	0.687	0.912
ESMC_600M, Gene_distance	0.877	0.83	0.853	0.857	0.826	0.849	0.712	0.912
ESMC_600M, PAAC	0.874	0.838	0.856	0.857	0.831	0.849	0.713	0.914
ESMC_600M, RSCU	0.86	0.837	0.849	0.85	0.829	0.843	0.699	0.918
ESMC_600M, CT, Copy_number	0.88	0.858	0.869	0.872	0.856	0.866	0.742	0.915
ESMC_600M, CT, DPC	0.857	0.829	0.843	0.847	0.824	0.838	0.692	0.906
ESMC_600M, CT, Euclidean_distance	0.885	0.844	0.865	0.869	0.848	0.864	0.736	0.91
ESMC_600M, CT, Expression	0.876	0.853	0.865	0.869	0.845	0.86	0.734	0.919
ESMC_600M, CT, Gene_distance	0.88	0.84	0.86	0.863	0.839	0.857	0.724	0.91
ESMC_600M, CT, PAAC	0.88	0.846	0.863	0.866	0.843	0.86	0.729	0.911
ESMC_600M, CT, RSCU	0.88	0.87	0.875	0.878	0.866	0.871	0.753	0.924
ESMC_600M, CT, RSCU, Copy_number	0.88	0.87	0.875	0.879	0.866	0.872	0.753	0.926
ESMC_600M, CT, RSCU, DPC	0.868	0.853	0.861	0.863	0.845	0.855	0.722	0.917
ESMC_600M, CT, RSCU, Euclidean_distance	0.88	0.865	0.872	0.875	0.86	0.868	0.748	0.925
ESMC_600M, CT, RSCU, Expression	0.851	0.849	0.85	0.854	0.836	0.843	0.702	0.914
ESMC_600M, CT, RSCU, Gene_distance	0.88	0.863	0.871	0.875	0.861	0.869	0.747	0.923
ESMC_600M, CT, RSCU, PAAC	0.865	0.84	0.852	0.857	0.835	0.848	0.709	0.918
ESMC_600M, CT, RSCU, Copy_number, DPC	0.863	0.854	0.859	0.863	0.843	0.851	0.722	0.924
ESMC_600M, CT, RSCU, Copy_number, Euclidean_distance	0.88	0.864	0.872	0.875	0.861	0.869	0.748	0.927
ESMC_600M, CT, RSCU, Copy_number, Expression	0.851	0.843	0.847	0.85	0.831	0.84	0.696	0.918
ESMC_600M, CT, RSCU, Copy_number, Gene_distance	**0.885**	**0.87**	**0.878**	**0.882**	**0.867**	**0.875**	**0.76**	**0.926**
ESMC_600M, CT, RSCU, Copy_number, PAAC	0.86	0.845	0.852	0.857	0.841	0.849	0.709	0.917
ESMC_600M, CT, RSCU, Copy_number, Gene_distance, DPC	0.871	0.859	0.865	0.869	0.851	0.86	0.735	0.923
ESMC_600M, CT, RSCU, Copy_number, Gene_distance, Euclidean_distance	0.885	0.87	0.878	0.882	0.867	0.875	0.76	0.926
ESMC_600M, CT, RSCU, Copy_number, Gene_distance, Expression	0.865	0.835	0.85	0.854	0.829	0.846	0.703	0.917
ESMC_600M, CT, RSCU, Copy_number, Gene_distance, PAAC	0.868	0.84	0.854	0.857	0.836	0.851	0.71	0.918
ESMC_600M, CT, RSCU, Copy_number, Gene_distance, Euclidean_distance, DPC	0.871	0.859	0.865	0.869	0.851	0.86	0.735	0.923
ESMC_600M, CT, RSCU, Copy_number, Gene_distance, Euclidean_distance, Expression	0.865	0.835	0.85	0.854	0.829	0.846	0.703	0.917
ESMC_600M, CT, RSCU, Copy_number, Gene_distance, Euclidean_distance, PAAC	0.868	0.842	0.855	0.857	0.836	0.85	0.711	0.919
ESMC_600M, CT, RSCU, Copy_number, Gene_distance, Euclidean_distance, DPC, Expression	0.866	0.839	0.852	0.857	0.837	0.849	0.71	0.912
ESMC_600M, CT, RSCU, Copy_number, Gene_distance, Euclidean_distance, DPC, PAAC	0.857	0.859	0.858	0.863	0.849	0.851	0.721	0.92
ESMC_600M, CT, RSCU, Copy_number, Gene_distance, Euclidean_distance, DPC, PAAC, Expression	0.859	0.847	0.853	0.857	0.839	0.847	0.711	0.918

a The feature combination with the highest F1 score is chosen. In case of a tie, the combination with a lesser number of feature groups is selected (shown in boldface).

**Table 3.** Regression: five-fold CV performance of XGBR model, trained on the Training set, using IFS with different feature combinations.^a^

Feature group	*R* ^2^	MSE	RMSE	MAE
Copy_number	0.276	0.478	0.689	0.47
CT	0.423	0.382	0.616	0.385
DPC	0.412	0.388	0.622	0.383
Euclidean_distance	0.059	0.625	0.789	0.562
Expression	0.399	0.4	0.626	0.422
Gene_distance	0.156	0.556	0.743	0.545
PAAC	0.434	0.373	0.609	0.383
RSCU	0.331	0.444	0.663	0.408
ESMC_600M	0.531	0.31	0.555	0.355
ESMC_600M, Copy_number	0.529	0.312	0.556	0.351
ESMC_600M, CT	0.51	0.324	0.568	0.36
ESMC_600M, DPC	0.492	0.336	0.577	0.366
ESMC_600M, Euclidean_distance	0.525	0.314	0.559	0.358
ESMC_600M, Expression	0.521	0.316	0.56	0.352
ESMC_600M, Gene_distance	0.53	0.312	0.556	0.352
ESMC_600M, PAAC	0.494	0.334	0.575	0.366
ESMC_600M, RSCU	0.517	0.319	0.563	0.354
ESMC_600M, Gene_distance, Copy_number	0.526	0.314	0.558	0.354
ESMC_600M, Gene_distance, CT	0.507	0.326	0.569	0.356
ESMC_600M, Gene_distance, DPC	0.489	0.338	0.579	0.369
ESMC_600M, Gene_distance, Euclidean_distance	0.53	0.311	0.556	0.353
ESMC_600M, Gene_distance, Expression	0.521	0.316	0.56	0.352
ESMC_600M, Gene_distance, PAAC	0.495	0.333	0.574	0.364
ESMC_600M, Gene_distance, RSCU	0.517	0.319	0.563	0.358
ESMC_600M, Gene_distance, Euclidean_distance, Copy_number	0.528	0.313	0.557	0.353
ESMC_600M, Gene_distance, Euclidean_distance, CT	0.505	0.327	0.57	0.356
ESMC_600M, Gene_distance, Euclidean_distance, DPC	0.489	0.339	0.579	0.37
ESMC_600M, Gene_distance, Euclidean_distance, Expression	0.522	0.316	0.56	0.352
ESMC_600M, Gene_distance, Euclidean_distance, PAAC	0.494	0.334	0.576	0.363
ESMC_600M, Gene_distance, Euclidean_distance, RSCU	0.52	0.317	0.561	0.358
ESMC_600M, Gene_distance, Euclidean_distance, Copy_number, CT	0.511	0.323	0.566	0.354
ESMC_600M, Gene_distance, Euclidean_distance, Copy_number, DPC	0.498	0.332	0.574	0.363
ESMC_600M, Gene_distance, Euclidean_distance, Copy_number, Expression	**0.531**	**0.31**	**0.554**	**0.347**
ESMC_600M, Gene_distance, Euclidean_distance, Copy_number, PAAC	0.503	0.328	0.57	0.362
ESMC_600M, Gene_distance, Euclidean_distance, Copy_number, RSCU	0.518	0.318	0.561	0.356
ESMC_600M, Gene_distance, Euclidean_distance, Copy_number, Expression, CT	0.503	0.328	0.57	0.352
ESMC_600M, Gene_distance, Euclidean_distance, Copy_number, Expression, DPC	0.498	0.332	0.574	0.362
ESMC_600M, Gene_distance, Euclidean_distance, Copy_number, Expression, PAAC	0.497	0.332	0.573	0.361
ESMC_600M, Gene_distance, Euclidean_distance, Copy_number, Expression, RSCU	0.523	0.315	0.559	0.351
ESMC_600M, Gene_distance, Euclidean_distance, Copy_number, Expression, RSCU, CT	0.52	0.317	0.561	0.35
ESMC_600M, Gene_distance, Euclidean_distance, Copy_number, Expression, RSCU, DPC	0.499	0.331	0.573	0.363
ESMC_600M, Gene_distance, Euclidean_distance, Copy_number, Expression, RSCU, PAAC	0.499	0.33	0.571	0.36
ESMC_600M, Gene_distance, Euclidean_distance, Copy_number, Expression, RSCU, CT, DPC	0.5	0.33	0.572	0.361
ESMC_600M, Gene_distance, Euclidean_distance, Copy_number, Expression, RSCU, CT, PAAC	0.517	0.32	0.562	0.35
ESMC_600M, Gene_distance, Euclidean_distance, Copy_number, Expression, RSCU, CT, DPC, PAAC	0.508	0.324	0.567	0.354

a The feature combination with the highest *R*^2^ is chosen (shown in boldface).

### 3.2 Model selection results

For both classification and regression tasks, selections of base and meta learners were conducted as described in Section 2.6. The results of IMI are presented in Tables 4 (for classification) and 5 (for regression). We used the models that were listed in Section 2.6. In Tables 4 and 5, only the top MI score–holding model combinations from each round of IMI are presented. The complete table is available in Supplementary Material, available as supplementary data at *Bioinformatics Advances* online. The model combinations “KNN” and “DTR and XGBR” had the highest MI scores (0.3878 and 0.7521, respectively) in Tables 4 (for classification) and 5 (for regression), respectively. So, KNN was selected to be the base learner for our classification model, and DTR and XGBR were chosen as the base learners for our regression model.

**Table 4.** Classification: highest MI scores of the model combinations in each round.^a^

Model combination	MI
KNN	**0.3878**
KNN, RF	0.3343
KNN, RF, SVM	0.3645
KNN, RF, SVM, GPC	0.3415
KNN, RF, SVM, GPC, ADA	0.3165
KNN, RF, SVM, GPC, ADA, XGB	0.3129
KNN, RF, SVM, GPC, ADA, XGB, LBM	0.3107
KNN, RF, SVM, GPC, ADA, XGB, LBM, MLP	0.2988
KNN, RF, SVM, GPC, ADA, XGB, LBM, MLP, GBC	0.2955
KNN, RF, SVM, GPC, ADA, XGB, LBM, MLP, GBC, QDA	0.2768

a The models were trained on the Training-60 set and evaluated on the Training-40 set. The model combination with the highest MI score is chosen (shown in boldface).

**Table 5.** Regression: highest MI scores of the model combinations in each round.^a^

Model combination	MI
DTR	0.7459
DTR, XGBR	**0.7521**
DTR, XGBR, SVR	0.7219
DTR, XGBR, SVR, RFR	0.6816
DTR, XGBR, SVR, RFR, KNNR	0.6655
DTR, XGBR, SVR, RFR, KNNR, GBR	0.6501
DTR, XGBR, SVR, RFR, KNNR, GBR, Ridge	0.609
DTR, XGBR, SVR, RFR, KNNR, GBR, Ridge, BR	0.581
DTR, XGBR, SVR, RFR, KNNR, GBR, Ridge, BR, Lasso	0.5207

a The models were trained on the Training-60 set and evaluated on the Training-40 set. The model combination with the highest MI score is chosen (shown in boldface).

**Figure 3.**
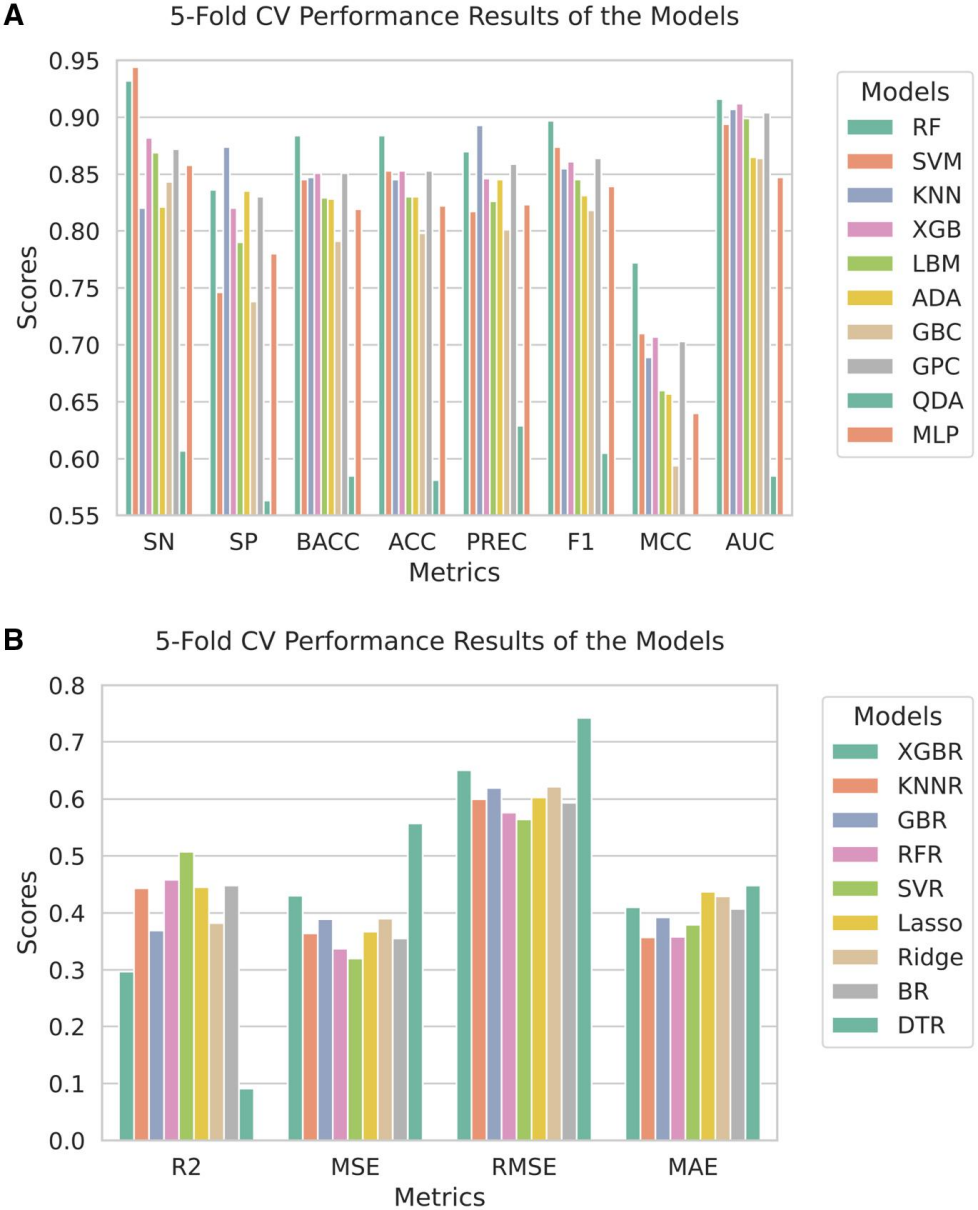
Meta model selection for classification and regression tasks. A) Five-fold CV performance of different models. All models were trained on the Training-40 set (classification). B) Five-fold CV performance of different models. All models were trained on the Training-40 set (regression).

The five-fold CV performance results for the meta learner selection are illustrated in Fig. 3. The reported values represent the average performance over all folds. In Fig. 3A (for classification), RF performed better than other models across most performance metrics, including F1 score, MCC, and AUC. So, RF was finalized as the meta learner for our classification model. SVR performed better than other models across most metrics (highest *R*^2^ score and lowest MSE and RMSE) in Fig. 3B (for regression). Therefore, SVR was chosen as the final meta learner for our regression model. To ensure more reliable and robust predictive results, we repeated the five-fold CV process 30× with different random seeds, averaging the results for both tasks. The results, which are displayed in Supplementary Fig. 2A and B, available as supplementary data at *Bioinformatics Advances* online, remained consistent with the single five-fold CV results, confirming the generalizability of our findings.

Thus, we finalized the stacking ensemble models for the classification and regression tasks. For classification, the finalized ensemble model is referred to as NFEmbed-C, and for regression it is referred to as NFEmbed-R.

### 3.3 Comparison with the state-of-the-art models

We compared our models with the state-of-the-art (SOTA) models in Tables 6 and 7. All models were trained on the Training set and evaluated on the Test set. In Table 6 (classification), our classification model NFEmbed-C outperformed Carmna across almost all performance metrics. NFEmbed-C achieved 0.949 sensitivity, 0.891 balanced accuracy, 0.892 F1 score, and 0.784 MCC on the Test set. Although Carmna attains a slightly higher test AUC (0.937 *vs.* 0.922), NFEmbed-C delivers higher test performance on the majority of metrics, including SN (0.949 *vs.* 0.872), BACC (0.891 *vs.* 0.853), ACC (0.889 *vs.* 0.852), PREC (0.841 *vs.* 0.830), F1 (0.892 *vs.* 0.850), and MCC (0.784 *vs.* 0.705), with the same SP (0.833). These gains reflect better overall classification quality at a fixed operating threshold. In Table 7 (regression), our regression model NFEmbed-R outperformed Carmna across all performance metrics. NFEmbed-R achieved 0.783 *R*^2^ score, 0.158 MSE, 0.398 RMSE, and 0.234 MAE on the Test set.

**Table 6.** Comparison with the state-of-the-art model for classification.^a^

Model	SN	SP	BACC	ACC	PREC	F1	MCC	AUC
Carmna	0.872	**0.833**	0.853	0.852	0.83	0.85	0.705	**0.937**
NFEmbed-C	**0.949**	**0.833**	**0.891**	**0.889**	**0.841**	**0.892**	**0.784**	0.922

a All models were trained on the Training set and evaluated on the Test set. The boldfaced values indicate the best performance for each evaluation metric.

**Table 7.** Comparison with the state-of-the-art model for regression.^a^

Model	*R* ^2^	MSE	RMSE	MAE
Carmna	0.557	0.323	0.569	0.335
NFEmbed-R	**0.783**	**0.158**	**0.398**	**0.234**

a All models were trained on the Training set and evaluated on the Test set. The boldfaced values indicate the best performance for each evaluation metric.

We conducted an ablation study, and the results are presented in Tables 8 (for classification) and 9 (for regression). Table 8 shows the difference in performance between the single RF model without BLP and the final stacking ensemble model (NFEmbed-C). The Training set was used to train both models, and the independent Test set was used to assess them. Similarly, in Table 9, the single SVR model without BLP and the final stacking ensemble model (NFEmbed-R) are compared in terms of performance. Both models were trained on the Training set and assessed on the Test set. In both tables, it is evident that the final stacking ensemble models outperformed their corresponding individual (non-ensemble) models. These results show the effectiveness of the stacking ensemble model architecture for both classification and regression tasks.

**Table 8.** Ablation study: performance comparison between the final stacking ensemble model (NFEmbed-C) and the single RF model without base learner probabilities (BLP).^a^

Model	SN	SP	BACC	ACC	PREC	F1	MCC	AUC
NFEmbed-C	**0.949**	**0.833**	**0.891**	**0.889**	**0.841**	**0.892**	**0.784**	**0.922**
RF	0.872	**0.833**	0.853	0.852	0.829	0.85	0.705	0.911

a Both models were trained on the Training set and evaluated on the independent Test set. The boldfaced values indicate the best performance for each evaluation metric.

**Table 9.** Ablation study: performance comparison between the final stacking ensemble model (NFEmbed-R) and the single SVR model without base learner probabilities (BLP).^a^

Model	*R* ^2^	MSE	RMSE	MAE
NFEmbed-R	**0.783**	**0.158**	**0.398**	**0.234**
SVR	0.527	0.346	0.588	0.352

a Both models were trained on the Training set and evaluated on the independent Test set. The boldfaced values indicate the best performance for each evaluation metric.

Additionally, we also trained a simple feed-forward neural network (FNN) with our optimal set of feature groups on the Training sets of both predictions and tested on their respective Test sets. The FNN delivered subpar performance compared to NFEmbed-C, achieving 0.923 SN, 0.833 SP, 0.878 BACC, 0.877 ACC, 0.837 PREC, 0.878 F1, 0.757 MCC, and 0.932 AUC on the Test set. Also, FNN could not do well for the regression task as well, obtaining 0.512 *R*^2^, 0.356 MSE, 0.597 RMSE, and 0.382 MAE on the Test set, poorer performance than our proposed model, NFEmbed-R. This highlights the effectiveness of using a stacking ensemble of traditional ML models over deep learning (DL) models for this task.

**Figure 4.**
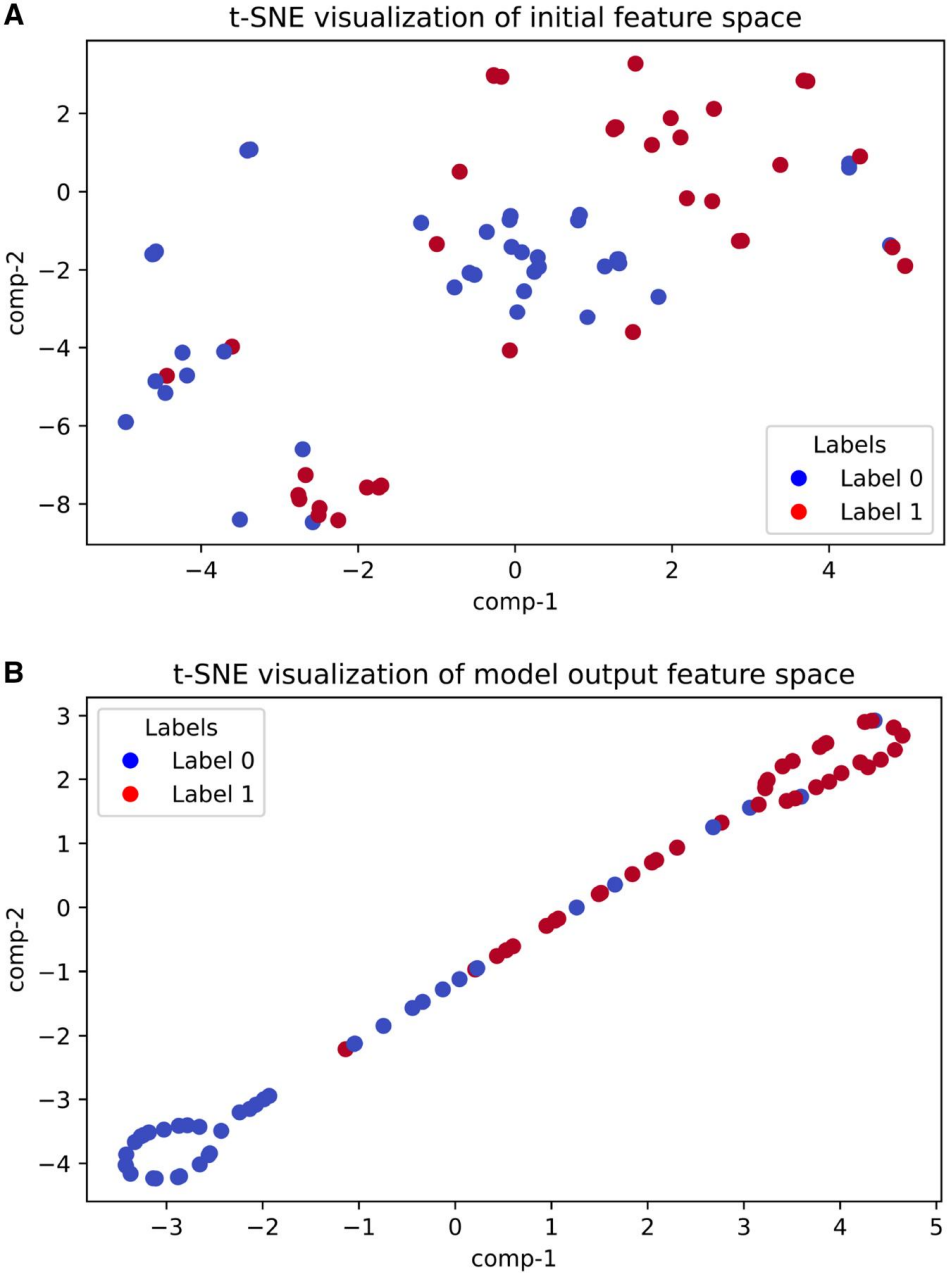
t-SNE plots for the proposed model, NFEmbed-C. A) The t-SNE visualization of the initial feature space. B) The t-SNE visualization of the model output feature space of the NFEmbed-C model on the Test dataset.

**Figure 5.**
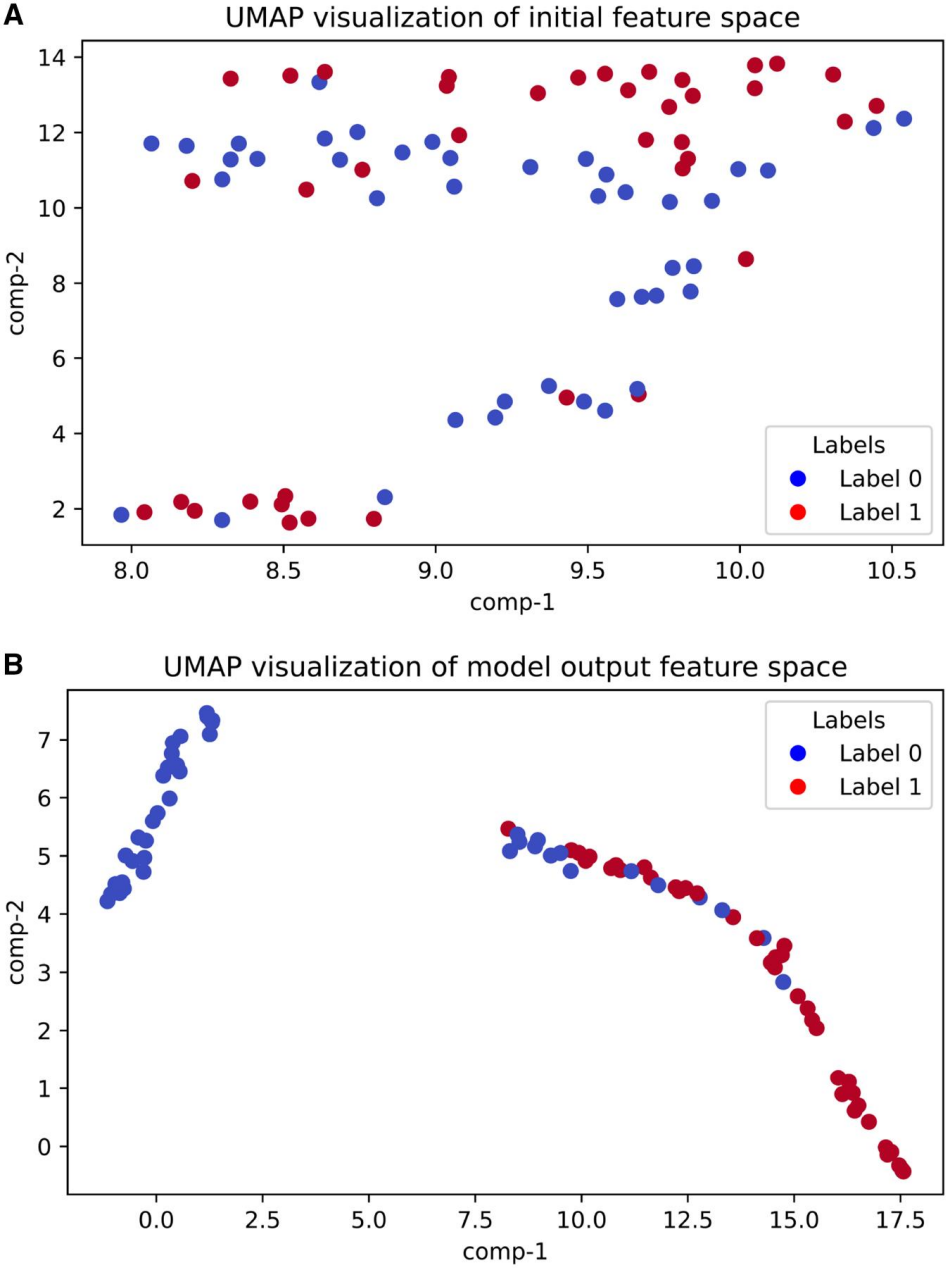
UMAP plots for the proposed model, NFEmbed-C. A) The UMAP visualization of the initial feature space. B) The UMAP visualization of the model output feature space of the NFEmbed-C model on the Test dataset.

**Figure 6.**
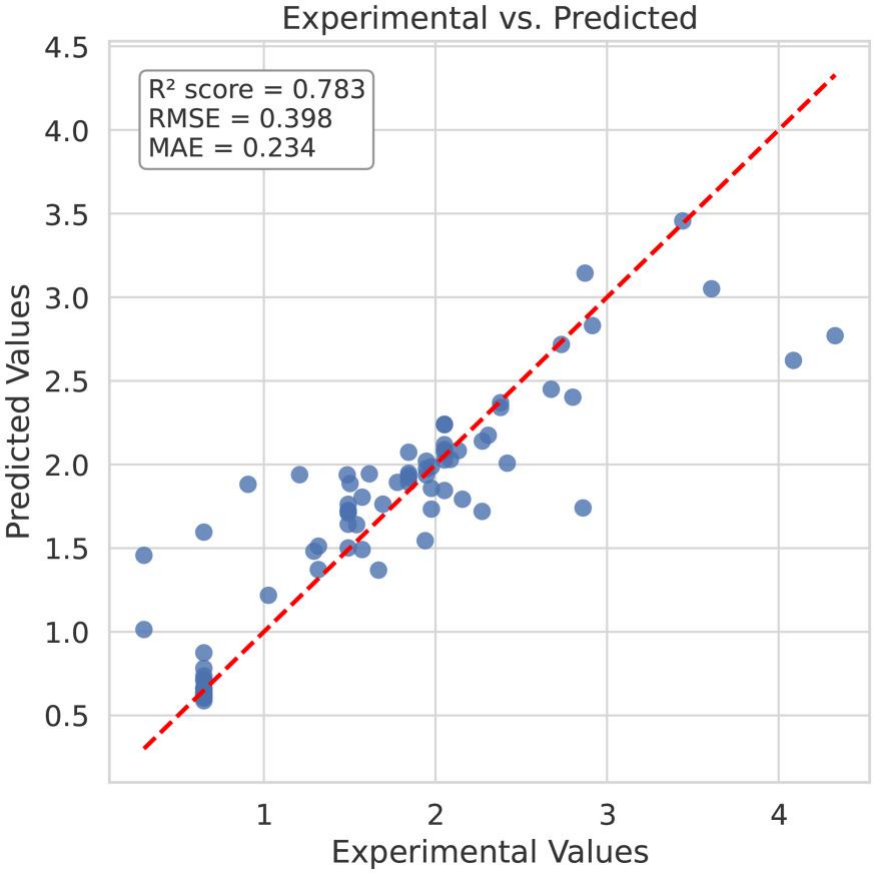
Regression plot showing the relationship between experimental and predicted values on the Test set of our proposal model, NFEmbed-R.

Figure 4 shows the t-SNE visualization on the feature space, while Fig. 5 shows the UMAP visualization. The initial feature space of the Test set samples is shown in the first plot of each figure, while the NFEmbed-C model’s output feature space is shown in the second plot. We can conclude from the figures that NFEmbed-C separates different class samples in a reasonable manner. It highlights the model’s capacity to identify discriminative patterns in the feature space and shows how well it can separate samples from different classes. In Fig. 6, the regression plot of NFEmbed-R on the Test set is demonstrated. The experimental and predicted values had a reasonably strong linear relationship, according to the regression plot. It shows that the model is doing a good job of identifying the underlying trends in the data. The points’ near alignment with the regression line indicates low error and high predictive accuracy.

### 3.4 Interpretability analysis using SHAP

**Figure 7.**
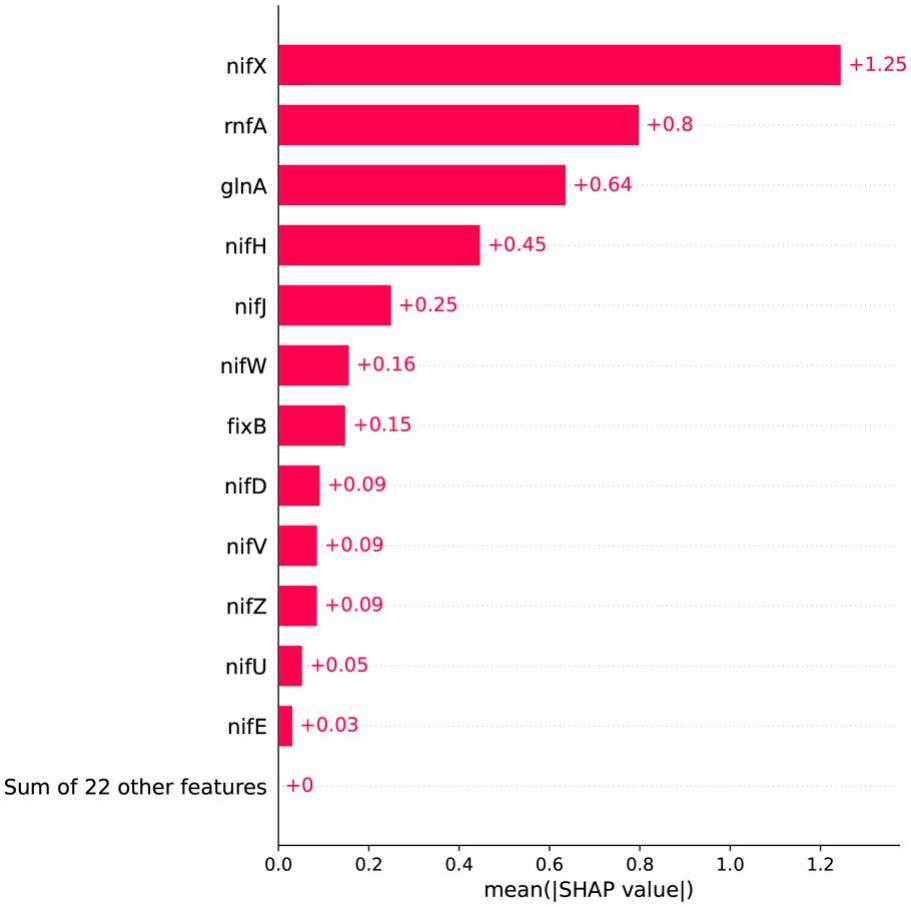
Analysis of the Copy_number feature group’s SHAP value that contributes to nitrogen fixation activity. The mean absolute SHAP values for each gene are displayed in a bar plot, which shows how important each gene is in the predictive model. The genes that contributed the most were nifX, rnfA, glnA, and nifH. Significant but smaller contributions are seen from accessory and structural genes such as fixB and nifJ. In the context of this model, the combined contribution of 22 additional genes is insignificant.

SHAP analysis of the Copy_number feature group is presented in Fig. 7. The mean absolute SHAP values for each gene are displayed in a bar plot. It shows how important each gene is in the predictive model. In that figure, the genes that contributed the most were nifX, rnfA, glnA, and nifH. nifX is involved in FeMo-co (Iron-Molybdenum Cofactor) synthesis. It accumulates an FeSMo-containing precursor (Rubio and Ludden 2005, Salinero-Lanzarote *et al.* 2025). rnfA is part of the Rnf complex that transfers low-potential electrons to nitrogenase via ferredoxin. Rnf complexes drive ferredoxin reduction and support nitrogen fixation (Kuhns *et al.* 2020, Alleman *et al.* 2022). A functional glnA in *Rhodopseudomonas capsulata* restored the normal ammonia-dependent repression of nitrogenase in glnA mutants, suggesting that glutamine synthetase (GS) activity controls the expression of the nif gene and signals nitrogen sufficiency (Scolnik *et al.* 1983, Jiang *et al.* 2023, Tang *et al.* 2023). The Fe protein is encoded by nifH, which also contributes to P-cluster and FeMo-co biosynthesis and moves electrons to the MoFe protein. It participates in stepwise metallocluster assembly as well as catalytic electron transfer (Rubio and Ludden 2005). As captured by our NFEmbed-C model, these findings highlight the roles of cofactor biosynthesis, electron transport, and nitrogen regulation genes in shaping nitrogenase activity.

**Figure 8.**
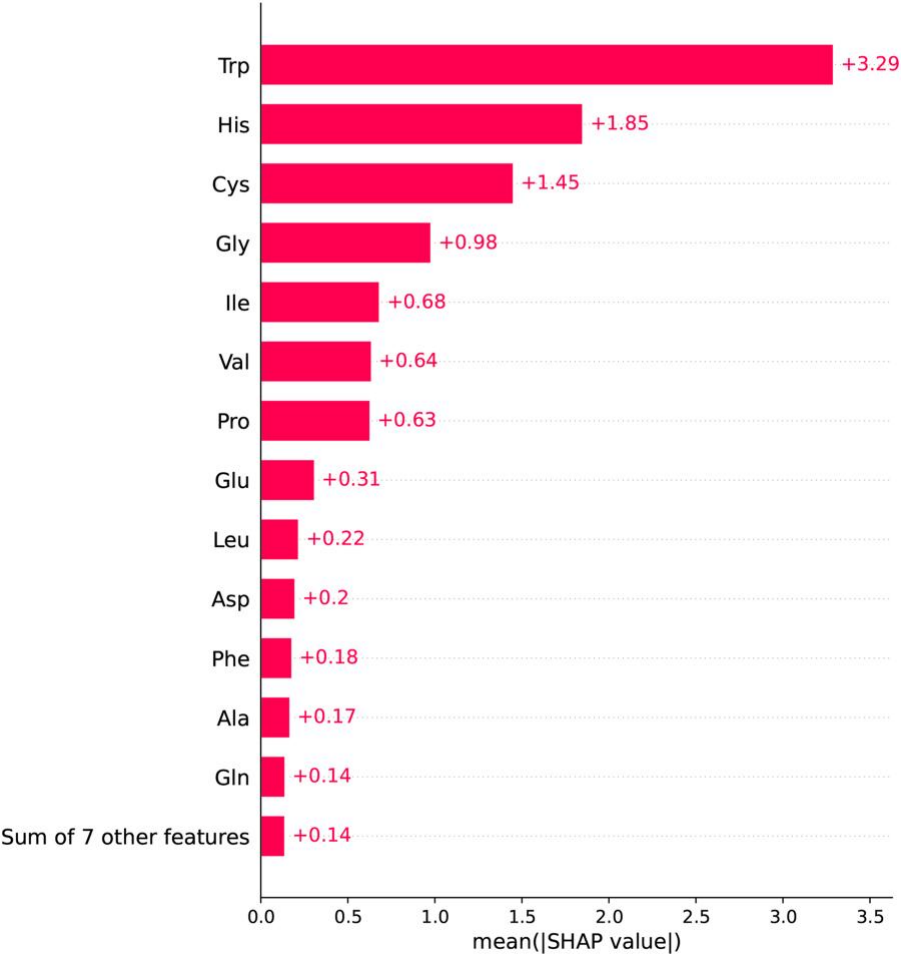
Analysis of the PAAC feature group’s SHAP value that contributes to nitrogen fixation activity. The mean absolute SHAP values for each amino acid are displayed in a bar plot, which shows how important each amino acid is in the predictive model. The amino acids that contributed the most were Trp, His, and Cys.

In Fig. 8, SHAP analysis of the PAAC feature group is presented. We trained the NFEmbed-C model with the additional feature group PAAC to get the SHAP contributions of the 20 AAs to the nitrogenase activity. The mean absolute SHAP values for each AA are displayed in a bar plot. It shows how important each AA is in the predictive model. The AAs that contribute the most are Trp, His, and Cys. Trp-α444 secures FeMo-co in its binding pocket by acting as a structural “lock”. Nitrogenase activity is drastically reduced, and FeMo-co insertion is greatly reduced when this large aromatic residue is mutated (Hu *et al.* 2006, Hu and Ribbe 2013). The FeMo-co is directly coordinated by His-442 and Cys-275 (Gu and Milton 2020, Salinero-Lanzarote *et al.* 2025), which bind to the iron and molybdenum atoms, respectively. It is critical for cofactor binding and functionality. They are highly conserved across nitrogenases (Garcia *et al.* 2020). These findings suggest that these results are closely linked to the roles of specific residues involved in FeMo-cofactor stabilization and electron transfer.

**Figure 9.**
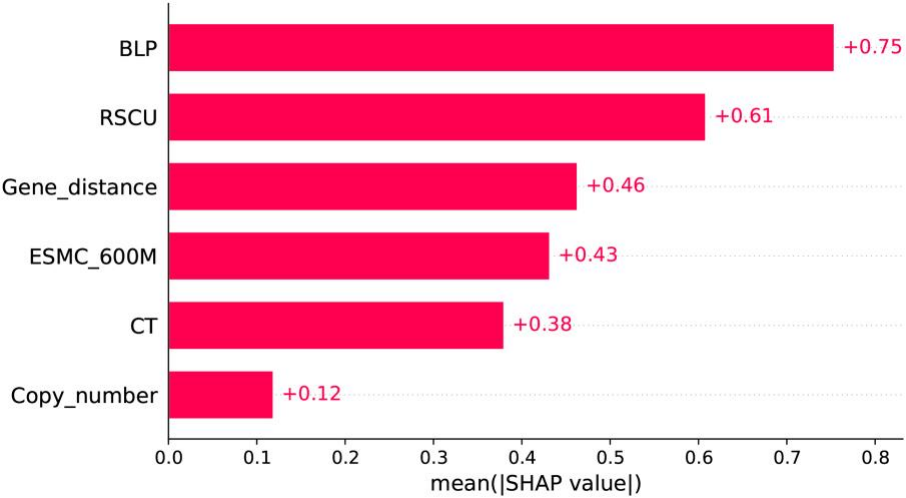
Analysis of six feature groups’ SHAP values that contribute to nitrogen fixation activity. The mean absolute SHAP values for each feature group used by the NFEmbed-C model are displayed in a bar plot. The feature group that contributed the most was BLP.

We also illustrated a bar plot in Fig. 9 where the mean absolute SHAP values for each feature group used in the NFEmbed-C model are shown. The mean absolute SHAP values for each feature group used by the NFEmbed-C model are displayed in a bar plot. Here, BLP contributed the most. It affirmed the effectiveness of the stacking ensemble architecture for the prediction task.

## 4 Discussion

In this study, we proposed two stacking ensemble models. One is NFEmbed-C (for classification) and the other is NFEmbed-R (for regression). Firstly, we explored different PLM embeddings and chose the most suitable PLM embeddings (ESMC_600M embeddings) for the prediction tasks. Then, we employed IFS on the feature groups to select the optimal feature group combination for the two proposed models. We chose “ESMC_600M, CT, RSCU, Copy_number, Gene_distance” for the NFEmbed-C model (classification) and “ESMC_600M, Gene_distance, Euclidean_distance, Copy_number, Expression” for the NFEmbed-R model (regression). For the model selection, several classification and regression models were analysed, and among them most suitable base and meta learners were chosen. Based on the MI scores of the IMI approach, we selected “KNN” and “DTR and XGBR” as the base learners for NFEmbed-C and NFEmbed-R models, respectively. After that, RF and SVR were chosen as the meta learner for NFEmbed-C and NFEmbed-R models, respectively, based on the five-fold CV performance results. Thus, the proposed stacking ensemble model was finalized. We illustrated t-SNE, UMAP visualizations on the feature space, along with regression plots to demonstrate the effectiveness of the proposed models. Furthermore, an interoperability analysis was done using SHAP to provide some biological contexts to the prediction of our proposed models. Ablation studies and SHAP analysis also supported the effectiveness of the stacking ensemble architecture of this prediction task. NFEmbed-C and NFEmbed-R outperformed the existing SOTA methods on the Test set. On the Test set, NFEmbed-C obtained a sensitivity of 0.949, balanced accuracy of 0.891, F1 score of 0.892, and MCC of 0.784, and NFEmbed-R obtained a 0.783 *R*^2^ score, 0.158 MSE, 0.398 RMSE, and 0.234 MAE.

The SHAP analysis of gene-level features (Copy_number group) reinforces the biological relevance of the NFEmbed-C model. Genes such as nifX, rnfA, glnA, and nifH were identified as top contributors to nitrogenase activity prediction. These genes are well-documented in the literature for their involvement in FeMo-co biosynthesis, electron transport, and nitrogen regulation—central processes in nitrogen fixation(Gu and Milton 2020, Jiang *et al.* 2023, Salinero-Lanzarote *et al.* 2025). The alignment between model-derived feature importance and known functional roles not only supports the interpretability of our model but also demonstrates its potential to uncover meaningful biological insights. The AA-level SHAP analysis (PAAC group) similarly highlights residues (Trp, His, Cys) that are structurally and functionally essential to nitrogenase function, especially in FeMo-co binding and electron transfer (Hu and Ribbe 2013, Gu and Milton 2020, Salinero-Lanzarote *et al.* 2025). The ability of NFEmbed-C to assign high importance to these residues suggests that the model is sensitive to physicochemical patterns that align with mechanistic knowledge.

However, there are some limitations to our work. A primary limitation of the study is the lack of data. For this reason, we did not utilize complex DL model architectures such as Recurrent Neural Networks (RNNs), Long Short-Term Memory (LSTM) RNN, and Transformers that are good for sequential data. Given that even a relatively simple DL model like FNN fails to generalize well (described in Section 3.3), applying more complex DL models is not advisable in our setting. For DL models, like Feedforward Neural Networks (FNNs), to generalize effectively, a significant quantity of data is usually needed. Because DL models typically have a large number of parameters to learn, which leads to overfitting, they perform poorly on small datasets (Goodfellow *et al.* 2016). In many recent bioinformatics studies, traditional ML (not DL) models have performed well (Liu *et al.* 2024, Rukh *et al.* 2024, Tran and Le 2024, Uddin *et al.* 2024, Yadav *et al.* 2024). Given the limited availability of nitrogenase activity data, constructing an additional independent dataset is not feasible. Future studies can focus on working with a large amount of data and use complex DL architectures for the prediction task. We also did not try to fine-tune PLMs for the prediction tasks because fine-tuning such large models (e.g. 600M+ parameters) on limited data risks overfitting and poor generalization. Also, due to computational constraints, fine-tuning these large models was not feasible within our available resources. To balance performance and practicality under these limitations, we decided to use the frozen embeddings from PLMs together with traditional ML models. If larger annotated datasets and more computational resources become available, future studies might investigate fine-tuning.

We did not employ any sampling approach (undersampling or oversampling) to the dataset in this study because of the Training and Test sets were overall balanced and did not need any additional sampling approach. In Section 3.1, we can see that ESMC_6B embeddings could not outperform ESMC_600M embeddings, despite having a larger feature size. This may be attributed to insufficient training data for the model utilizing the larger feature set to generalize effectively. We acknowledge the risk of overfitting given the relatively small dataset size (402 samples) and the high dimensionality of PLM-derived embeddings. To address this, we employed five-fold CV throughout to ensure the generalizability of the results and repeated the five-fold CV process 30× using different random seeds to mitigate performance fluctuations due to random data splits. These repetitions allowed us to assess the stability and consistency of model performance, helping identify embeddings that consistently performed well (e.g. ESMC 600M), and those that likely overfit (e.g. ESMC 6B), despite larger embedding sizes. In Section 3.4, the mean absolute SHAP values were lower for ESMC_600M embeddings than those of the feature groups: RSCU and Gene_distance. This is because we averaged over the column dimension to compute the mean values. However, if feature dimensionality is taken into account, the ESMC_600M embeddings would exhibit higher overall absolute SHAP values than the other feature groups.

The use of SHAP analysis was motivated by our goal in this study, which was to comprehend the global contributions of features to the model’s predictions. A dataset-wide view of how individual features affect model behavior is made achievable by SHAP, which aggregates feature importance across all samples. On the other hand, LIME (Garreau and Luxburg 2020) approximates the model’s decision boundary around a single instance to produce local explanations. SHAP was a better fit for our analysis because of our emphasis on global interpretability and dataset-wide feature contributions.

We acknowledge the potential of using structural features from AlphaFold (Jumper *et al.* 2021) predicted structures, but we will leave that for future studies. We kept a single meta learner within the stacking ensemble model architecture for this study in light of the findings and standard practice. However, investigating multiple meta learners might also be an area for further research.

NFEmbed-C and NFEmbed-R significantly improve the task of nitrogenase activity prediction. The models and related codes can be found at https://github.com/nafcoder/NFEmbed as open-source scripts. Findings from our study should help guide the selection and development of high-efficiency nitrogen-fixing bacterial strains and encourage advances in the production and application of biofertilizers.

## Supplementary Material

vbaf204_Supplementary_Data

## Data Availability

Datasets, NFEmbed model, and scripts to reproduce the results are available at https://github.com/nafcoder/NFEmbed.
