# Supplementary material for: NFEmbed: modeling nitrogenase activity via classification and regression with pretrained protein embeddings
Source: Bioinform Adv. 2025 Aug 23;5(1):vbaf204. doi: 10.1093/bioadv/vbaf204 (PMC12417089; doi:10.1093/bioadv/vbaf204)
Supplement: vbaf204_Supplementary_Data [file vbaf204_supplementary_data.zip › OUP_NFEmbed_Supplementary.pdf]

Supplementary information: NFEmbed: Modeling Nitrogenase Activity via Classification and Regression with Pretrained Protein Embeddings

Md Muhaiminul Islam Nafi[1, 2, 3] and Abdullah Al Mohaimin[1]

[1]Department of CSE, BUET, Dhaka 1000, Bangladesh
[2]Department of CSE, United International University (UIU), Dhaka 1212, Bangladesh
[3]Corresponding author: Tel: +8801704953445, Email: nafiislam964@gmail.com

August 20, 2025

Supplementary Material

This supplementary section provides additional tables and figures.

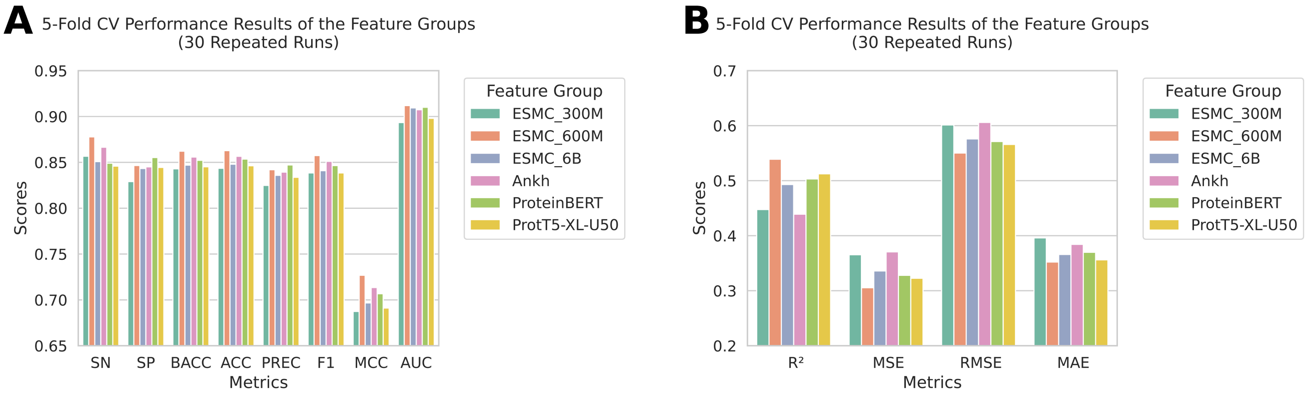

Figure S1: First step of feature selection for classification and regression tasks, averaged over 30 repeated runs with different random seeds. **A)** 5-Fold CV performance of different XGB models using six distinct protein language model embeddings, averaged over 30 repeated runs with different random seeds. All models were trained on the Training set. (classification) **B)** 5-Fold CV performance of different XGBR models using six distinct protein language model embeddings, averaged over 30 repeated runs with different random seeds. All models were trained on the Training set. (regression)

Table S1: Classification: 5-Fold CV performance of XGB model, averaged over 30 repeated runs with different random seeds, trained on the Training set, using IFS with different feature combinations. The feature combination with the highest F1 score is chosen. In case of a tie, the combination with a lesser number of feature groups is selected (shown in boldface).

**Feature Group**	**SN**	**SP**	**BACC**	**ACC**	**PREC**	**F1**	**MCC**	**AUC**
Copy_number	0.789	0.733	0.761	0.763	0.729	0.756	0.525	0.833
CT	0.853	0.849	0.851	0.851	0.837	0.843	0.702	0.908
DPC	0.859	0.859	0.859	0.862	0.854	0.854	0.724	0.917
Euclidean_distance	0.707	0.7	0.703	0.701	0.69	0.691	0.41	0.781
Expression	0.794	0.826	0.81	0.811	0.808	0.799	0.62	0.886
Gene_distance	0.681	0.653	0.667	0.666	0.644	0.659	0.334	0.751
PAAC	0.866	0.86	0.863	0.863	0.851	0.856	0.728	0.903
RSCU	0.804	0.848	0.826	0.827	0.829	0.815	0.653	0.896
ESMC_600M	0.878	0.847	0.862	0.863	0.842	0.857	0.727	0.912
ESMC_600M,Copy_number	0.866	0.853	0.859	0.861	0.848	0.855	0.722	0.911
ESMC_600M,CT	0.875	0.853	0.864	0.866	0.85	0.861	0.731	0.905
ESMC_600M,DPC	0.879	0.841	0.86	0.861	0.838	0.856	0.722	0.911
ESMC_600M,Euclidean_distance	0.872	0.855	0.864	0.865	0.85	0.859	0.73	0.912
ESMC_600M,Expression	0.864	0.853	0.858	0.859	0.846	0.853	0.72	0.915
ESMC_600M,Gene_distance	0.876	0.843	0.86	0.861	0.839	0.855	0.722	0.909
ESMC_600M,PAAC	0.878	0.843	0.861	0.862	0.841	0.857	0.725	0.912
ESMC_600M,RSCU	0.866	0.848	0.857	0.858	0.842	0.852	0.716	0.913
ESMC_600M,CT,Copy_number	0.874	0.856	0.865	0.867	0.851	0.862	0.733	0.907
ESMC_600M,CT,DPC	0.871	0.849	0.86	0.862	0.844	0.856	0.724	0.908
ESMC_600M,CT,Euclidean_distance	0.875	0.853	0.864	0.866	0.85	0.861	0.731	0.906
ESMC_600M,CT,Expression	0.84	0.83	0.835	0.837	0.824	0.83	0.673	0.892
ESMC_600M,CT,Gene_distance	0.87	0.857	0.863	0.865	0.852	0.86	0.73	0.906
ESMC_600M,CT,PAAC	0.869	0.853	0.861	0.862	0.847	0.857	0.723	0.907
ESMC_600M,CT,RSCU	0.879	0.86	0.869	0.871	0.856	0.866	0.741	0.914
ESMC_600M,CT,RSCU,Copy_number	0.88	0.86	0.87	0.872	0.856	0.867	0.742	0.912
ESMC_600M,CT,RSCU,DPC	0.868	0.866	0.867	0.869	0.859	0.862	0.736	0.914
ESMC_600M,CT,RSCU,Euclidean_distance	0.879	0.858	0.868	0.87	0.854	0.865	0.739	0.914
ESMC_600M,CT,RSCU,Expression	0.867	0.863	0.865	0.866	0.854	0.859	0.73	0.912
ESMC_600M,CT,RSCU,Gene_distance	0.854	0.813	0.834	0.835	0.81	0.831	0.67	0.895
ESMC_600M,CT,RSCU,PAAC	0.87	0.852	0.861	0.863	0.846	0.857	0.724	0.91
ESMC_600M,CT,RSCU,Copy_number,DPC	0.868	0.871	0.87	0.872	0.863	0.864	0.743	0.917
ESMC_600M,CT,RSCU,Copy_number,Euclidean_distance	0.88	0.858	0.869	0.87	0.854	0.866	0.74	0.913
ESMC_600M,CT,RSCU,Copy_number,Expression	0.863	0.862	0.863	0.864	0.852	0.857	0.726	0.913
ESMC_600M,CT,RSCU,Copy_number,Gene_distance	**0.883**	**0.862**	**0.873**	**0.875**	**0.859**	**0.87**	**0.748**	**0.912**
ESMC_600M,CT,RSCU,Copy_number,PAAC	0.881	0.86	0.87	0.872	0.855	0.867	0.742	0.913
ESMC_600M,CT,RSCU,Copy_number,Gene_distance,DPC	0.871	0.873	0.872	0.874	0.866	0.867	0.748	0.917
ESMC_600M,CT,RSCU,Copy_number,Gene_distance,Euclidean_distance	0.883	0.862	0.873	0.875	0.859	0.87	0.748	0.912
ESMC_600M,CT,RSCU,Copy_number,Gene_distance,Expression	0.87	0.859	0.864	0.866	0.852	0.86	0.731	0.913
ESMC_600M,CT,RSCU,Copy_number,Gene_distance,PAAC	0.88	0.862	0.871	0.872	0.857	0.867	0.743	0.913
ESMC_600M,CT,RSCU,Copy_number,Gene_distance,Euclidean_distance,DPC	0.871	0.873	0.872	0.874	0.866	0.867	0.748	0.917
ESMC_600M,CT,RSCU,Copy_number,Gene_distance,Euclidean_distance,Expression	0.87	0.859	0.864	0.866	0.852	0.86	0.731	0.913
ESMC_600M,CT,RSCU,Copy_number,Gene_distance,Euclidean_distance,PAAC	0.88	0.863	0.871	0.872	0.857	0.867	0.743	0.913
ESMC_600M,CT,RSCU,Copy_number,Gene_distance,Euclidean_distance,DPC,Expression	0.872	0.866	0.869	0.872	0.862	0.865	0.743	0.912
ESMC_600M,CT,RSCU,Copy_number,Gene_distance,Euclidean_distance,DPC,PAAC	0.863	0.869	0.866	0.868	0.862	0.861	0.736	0.913
ESMC_600M,CT,RSCU,Copy_number,Gene_distance,Euclidean_distance,DPC,PAAC,Expression	0.859	0.87	0.865	0.867	0.862	0.858	0.733	0.915

Table S2: Regression: 5-Fold CV performance of XGBR model, averaged over 30 repeated runs with different random seeds, trained on the Training set, using IFS with different feature combinations. The feature combination with the highest R^2 is chosen (shown in boldface).

**Feature Group**	$\mathbf{R^2}$	**MSE**	**RMSE**	**MAE**
Copy_number	0.301	0.462	0.678	0.461
CT	0.44	0.37	0.605	0.379
DPC	0.438	0.37	0.607	0.373
Euclidean_distance	0.077	0.611	0.779	0.553
Expression	0.382	0.41	0.637	0.434
Gene_distance	0.16	0.555	0.742	0.536
PAAC	0.46	0.357	0.596	0.377
RSCU	0.324	0.447	0.666	0.412
ESMC_600M	0.539	0.305	0.55	0.352
ESMC_600M, Copy_number	0.533	0.31	0.554	0.353
ESMC_600M, CT	0.519	0.32	0.563	0.358
ESMC_600M, DPC	0.521	0.317	0.56	0.355
ESMC_600M, Euclidean_distance	0.538	0.306	0.551	0.353
ESMC_600M, Expression	0.536	0.307	0.552	0.35
ESMC_600M, Gene_distance	0.54	0.305	0.55	0.349
ESMC_600M, PAAC	0.517	0.321	0.563	0.359
ESMC_600M, RSCU	0.532	0.31	0.554	0.352
ESMC_600M, Gene_distance, Copy_number	0.531	0.311	0.555	0.353
ESMC_600M, Gene_distance, CT	0.524	0.316	0.56	0.354
ESMC_600M, Gene_distance, DPC	0.52	0.318	0.561	0.357
ESMC_600M, Gene_distance, Euclidean_distance	0.54	0.305	0.55	0.349
ESMC_600M, Gene_distance, Expression	0.538	0.305	0.55	0.347
ESMC_600M, Gene_distance, PAAC	0.518	0.32	0.562	0.358
ESMC_600M, Gene_distance, RSCU	0.531	0.31	0.555	0.353
ESMC_600M, Gene_distance, Euclidean_distance, Copy_number	0.531	0.311	0.555	0.353
ESMC_600M, Gene_distance, Euclidean_distance, CT	0.523	0.317	0.56	0.354
ESMC_600M, Gene_distance, Euclidean_distance, DPC	0.52	0.318	0.561	0.357
ESMC_600M, Gene_distance, Euclidean_distance, Expression	0.502	0.33	0.571	0.391
ESMC_600M, Gene_distance, Euclidean_distance, PAAC	0.518	0.32	0.563	0.358
ESMC_600M, Gene_distance, Euclidean_distance, RSCU	0.495	0.334	0.576	0.398
ESMC_600M, Gene_distance, Euclidean_distance, Copy_number, CT	0.526	0.314	0.558	0.353
ESMC_600M, Gene_distance, Euclidean_distance, Copy_number, DPC	0.521	0.317	0.561	0.354
ESMC_600M, Gene_distance, Euclidean_distance, Copy_number, Expression	**0.542**	**0.304**	**0.548**	**0.347**
ESMC_600M, Gene_distance, Euclidean_distance, Copy_number, PAAC	0.527	0.314	0.558	0.353
ESMC_600M, Gene_distance, Euclidean_distance, Copy_number, RSCU	0.533	0.31	0.554	0.351
ESMC_600M, Gene_distance, Euclidean_distance, Copy_number, Expression, CT	0.519	0.32	0.562	0.353
ESMC_600M, Gene_distance, Euclidean_distance, Copy_number, Expression, DPC	0.521	0.317	0.561	0.355
ESMC_600M, Gene_distance, Euclidean_distance, Copy_number, Expression, PAAC	0.525	0.315	0.557	0.354
ESMC_600M, Gene_distance, Euclidean_distance, Copy_number, Expression, RSCU	0.531	0.311	0.555	0.351
ESMC_600M, Gene_distance, Euclidean_distance, Copy_number, Expression, RSCU, CT	0.524	0.316	0.559	0.351
ESMC_600M, Gene_distance, Euclidean_distance, Copy_number, Expression, RSCU, DPC	0.515	0.32	0.563	0.357
ESMC_600M, Gene_distance, Euclidean_distance, Copy_number, Expression, RSCU, PAAC	0.518	0.319	0.561	0.356
ESMC_600M, Gene_distance, Euclidean_distance, Copy_number, Expression, RSCU, CT, DPC	0.51	0.324	0.567	0.359
ESMC_600M, Gene_distance, Euclidean_distance, Copy_number, Expression, RSCU, CT, PAAC	0.521	0.318	0.561	0.35
ESMC_600M, Gene_distance, Euclidean_distance, Copy_number, Expression, RSCU, CT, PAAC, DPC	0.508	0.325	0.568	0.357

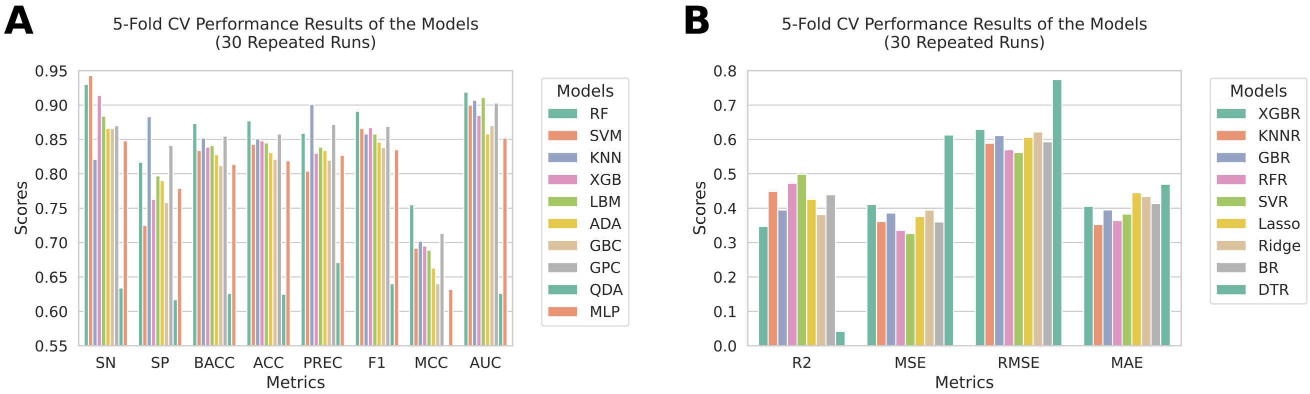

Figure S2: Meta model selection for classification and regression tasks, averaged over 30 repeated runs with different random seeds. **A)** 5-Fold CV performance of different models, averaged over 30 repeated runs with different random seeds. All models were trained on the Training-40 set. (classification) **B)** 5-Fold CV performance of different models, averaged over 30 repeated runs with different random seeds. All models were trained on the Training-40 set. (regression)
